# Discovery of New Everninomicin Analogs from a Marine-Derived *Micromonospora* sp. by Metabolomics and Genomics Approaches

**DOI:** 10.3390/md23080316

**Published:** 2025-07-31

**Authors:** Tae Hyun Lee, Nathan J. Brittin, Imraan Alas, Christopher D. Roberts, Shaurya Chanana, Doug R. Braun, Spencer S. Ericksen, Song Guo, Scott R. Rajski, Tim S. Bugni

**Affiliations:** 1 Pharmaceutical Sciences Division, University of Wisconsin—Madison, Madison, WI 53705, USA; taehyun.lee@wisc.edu (T.H.L.); nbrittin@wisc.edu (N.J.B.); imraanalas@gmail.com (I.A.); cdroberts3@wisc.edu (C.D.R.); shaurya.chanana@gmail.com (S.C.); drbraun1@wisc.edu (D.R.B.); scott.rajski@wisc.edu (S.R.R.); 2 Small Molecule Screening Facility, UW Carbone Cancer Center, Madison, WI 53792, USA; ssericksen@wisc.edu (S.S.E.); sguo6@wisc.edu (S.G.); 3 Lachman Institute for Pharmaceutical Development, University of Wisconsin—Madison, Madison, WI 53705, USA

**Keywords:** HCAPCA, Global Natural Products Social molecular networking (GNPS), genome mining, *Micromonospora*, antibacterial

## Abstract

During the course of genome mining initiatives, we identified a marine-derived *Micromonospora*, assigned here as strain WMMD956; the genome of WMMD956 appeared to contain a number of features associated with everninomicins, well-known antimicrobial orthosomycins. In addition, LCMS-based hierarchical clustering analysis and principal component analysis (*hcapca*) revealed that WMMD956 displayed an extreme degree of metabolomic and genomic novelty. Dereplication of high-resolution tandem mass spectrometry (HRMS/MS) and Global Natural Product Social molecular networking platform (GNPS) analysis of WMMD956 resulted in the identification of several analogs of the previously known everninomicin. Chemical structures were unambiguously confirmed by HR-ESI-MS, 1D and 2D NMR experiments, and the use of MS/MS data. The isolated metabolites, **1**–**3**, were evaluated for their antibacterial activity against methicillin-resistant *Staphylococcus aureus* (MRSA).

## 1. Introduction

The world’s oceans house a vast array of chemical and biological diversity. Accordingly, it is no surprise that marine-derived natural products represent incredible tools for the advancement of humanity, especially in the arena of global health. To date, more than 30,000 natural products of marine origin have been discovered, and these agents have made invaluable contributions to the advancement of human medicine [1,2]. Importantly, many of these compounds, like their terrestrial counterparts, were originally identified using conventional bioassay-guided fractionation approaches and impressively large compound/sample libraries. However, advances in genomics, metabolomics, and other technologies have enabled the efficient surveying of microbial genomes for genes of interest likely to translate to interesting chemistry. Coordinated with many of these advances have been intensified efforts to sequence the genomes of marine-derived microbes. Unsurprisingly, genome mining has become a powerful tool by which to discover novel natural products on the basis of the biosynthetic genes driving their production; this has especially been the case with marine organisms [3,4]. These and other “omics-based” methods of identifying new compounds hold tremendous potential to impact infectious disease therapies.

It is now widely acknowledged that infectious diseases, especially pan-drug-resistant ones, represent a dire threat to humanity on the global scale. This idea, though widely held for the last several decades, was truly validated with the COVID-19 pandemic. Beyond viral pathogens such as COVID-19, alarms have long been sounded with respect to bacterial pathogens; especially noteworthy have been the ESKAPE pathogens (*Enterococcus faecium*, *Staphylococcus aureus*, *Klebsiella pneumoniae*, *Acinetobacter baumannii*, *Pseudomonas aeruginosa*, and *Enterobacter* species), as well as fungal pathogens such as *Candida albicans* and *auris* [5]. Reports of these organisms developing resistance to established antimicrobials and their increasing prevalence, especially in clinical settings, have dramatically increased in the last decade, inspiring grave concern but fueling impressive innovations [6]. In particular, innovations in the arena of natural product discovery have been especially impressive and readily translate to the formulation of new antimicrobial therapeutic strategies. The establishment of the Natural Products Magnetic Resonance Database (NP-MRD), the Natural Products Atlas, the GNPS framework, and isotopic methods capable of linking natural products to their biosynthetic gene clusters—together with hcapca paradigms, chemical genomics assays, and a suite of emerging technologies that enable rapid correlation of genomics and metabolomics data, connect phenotypic changes to chemotypes, and leverage rapidly advancing artificial intelligence (AI) systems—has dramatically transformed the landscape of drug discovery over the past 5–10 years and is expected to continue driving innovation in the field. Herein, we describe our application of some of these tools (most prominently *hcapca*, GNPS networking, and genome mining) to a novel marine-derived *Micromonospora* sp. bacterium en route to the discovery of new everninomicin-like agents.

**Figure 1:**
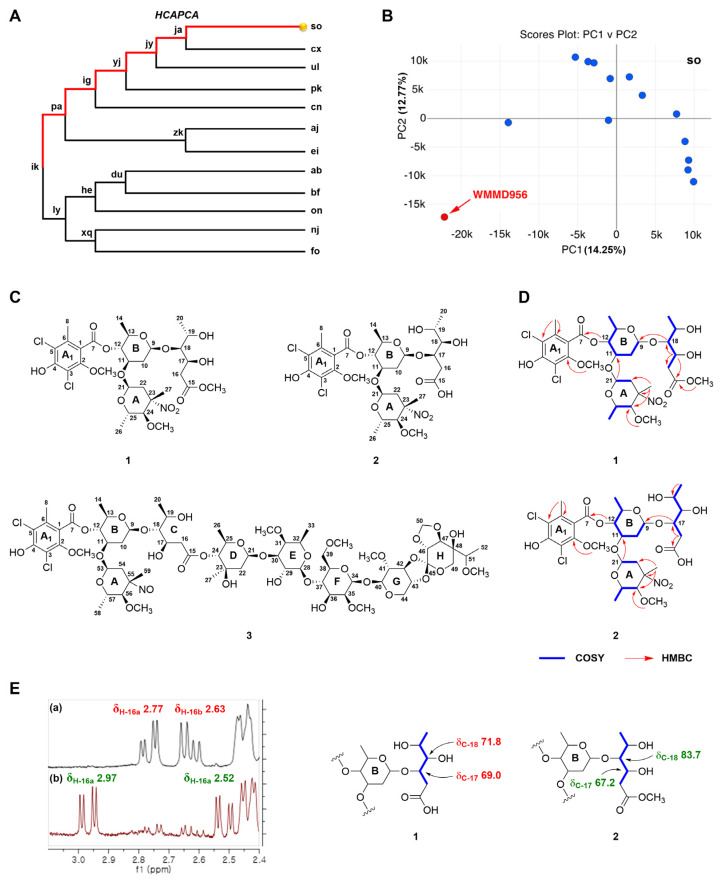
Strain prioritization from *Micromonospora* spp. bacteria and structural characterization of isolated compounds. (**A**) Partial dendrogram resulting from the HCA step of *hcapca* applied to 168 strain panel. (**B**) PCA scores plot of the subcluster “so” containing 14 marine-derived *Micromonospora* revealing high-variance strain WMMD956, as determined on the basis of metabolomic diversity. (**C**) Chemical structures of compounds **1**–**3**. (**D**) Key COSY (blue bold) and HMBC (red arrows) correlations for **1** and **2**. (**E**) The zoomed-in ^1^H NMR spectra of H-16 for **1** (**a**) and **2** (**b**) (**left**), as well as their distinctive differences regarding carbon chemical shift values (δ_C-17_ and δ_C-18_) (**right**).

In our efforts to discover structurally intriguing secondary metabolites produced from marine-derived *Micromonospora* sp. bacteria, *hcapca* and a genomic approach using antiSMASH were carried out on a panel of marine-derived bacteria. We initially conducted LC-MS-based hierarchical clustering analysis and principal component analysis (*hcapca*) on a pool of 168 marine bacteria; the intent was to identify metabolically unique strains [7]. Among the subclusters within the tree generated via hierarchical clustering analysis (HCA) was the subcluster “so”, containing 14 clustered strains, which indicated WMMD956—highlighted in red—was distinct from the other bacterial strains (Figure 1A). The PCA performed on the subcluster “so” revealed WMMD956 as the strongest metabolic outlier (Figure 1B), being easily differentiable from the nearest samples. Having identified WMMD956 via *hcapca* processing of the 168 member panel, we subsequently carried out antiSMASH processing of the WMMD956 genome [8]. This genome analysis revealed the presence of a biosynthetic gene cluster responsible for the biosynthesis of oligosaccharides with 100% similarity to reported gene cluster from this family (Figure S1, Supporting Information). Orthosomycins are a representative class of oligosaccharide antibiotics characterized by the presence of one or more orthoester moieties; many such NPs have been found to display significant activity against Gram-positive pathogens [9,10,11]. Consistent with these bioinformatics-informed findings, we carried out an initial activity screening using fractions A–E, produced from the acetone extract of WMMD956 against methicillin-resistant *Staphylococcus aureus* (MRSA) to attempt to obtain more highly refined antibiotic oligosaccharides likely related to the suspected orthosomycins. Notably, fractions A and D were found to exhibit moderate MRSA inhibition activities (Figure S2, Supporting Information), and it was from these fractions that compounds **1**–**3** were isolated (Figure 1C). It is worth noting that the antimicrobial activities noted at this juncture, along with the metabolomics and genomics findings for WMMD956, were sufficient to inspire structure isolation efforts.

## 2. Results and Discussion

Sporacin A (**1**) was isolated as a pale yellowish gum. The molecular formula of **1** was determined to be C_30_H_43_Cl_2_NO_15_ based on a sodiated molecular ion at *m*/*z* 750.1875 [M + Na]^+^ in the HRESIMS (calculated for C_30_H_43_Cl_2_NNaO_15_, 750.1907, error = 4.3 ppm) and an ammonium adducted molecular ion at *m*/*z* 745.2321 [M + NH_4_]^+^ in the HRESIMS (calculated for C_30_H_47_Cl_2_N_2_O_15_, 745.2353, error = 4.3 ppm). The ^1^H NMR and HSQC analysis for **1** revealed characteristic signals for one dichloroisoeverninic acid (DIA, ring A_1_) [δ_H_ 3.91 (3H, s, 2-OCH_3_) and 2.38 (3H, s, H-8); δ_C_ 166.2 (C-7), 153.7 (C-2), 134.4 (C-6), 116.4 (C-3), 119.2 (C-5), 114.2 (C-1), 61.1 (2-OCH_3_), and 17.0 (C-8)] and two sugar moieties (A/B) [δ_H_ 5.03 (1H, d, *J* = 4.4 Hz, H-21), 3.62 (1H, m, H-24), 3.50 (1H, m, H-25), 3.34 (3H, s, 24-OCH_3_), 2.43 (1H, d, *J* = 13.7 Hz, H-22a), 2.08 (1H, d, *J* = 13.6 Hz, H-22b), 1.69 (3H, s, H-27), and 0.77 (3H, d, *J* = 6.0 Hz, H-26); δ_C_ 92.8 (C-21), 89.8 (C-23), 84.4 (C-24), 65.9 (C-25), 54.7 (24-OCH_3_), 40.1 (C-22), 18.4 (C-27), and 16.4 (C-26) for A] and [δ_H_ 4.80 (1H, t, *J* = 9.4 Hz, H-12), 4.71(1H, d, *J* = 9.3 Hz, H-9), 3.99 (1H, m, H-11), 3.60 (1H, m, H-13), 2.45 (1H, m, H-10a), 1.61 (1H, m, H-10b), and 1.35 (3H, d, *J* = 6.0 Hz, H-14); δ_C_ 99.8 (C-9), 76.2 (C-12), 72.4 (C-11), 70.6 (C-13), 35.8 (C-10), and 17.3 (C-14) for B]. (Table 1). These analyses of **1** were consistent with those of L-evernitrose (A), DIA (A_1_), and D-olivose (B) of everninimicin H [12]. The major two differences were in the presence of an additional linear structure [δ_H_ 4.33 (1H, m, H-17), 3.95 (1H, m, H-19), 3.52 (1H, m, H-18), 2.77 (1H, dd, *J* = 16.3 and 4.9 Hz, H-16a), 2.63 (1H, dd, *J* = 16.2 and 8.0 Hz, H-16b), and 1.26 (1H, d, *J* = 6.2 Hz, H-20); δ_C_ 172.7 (C-15), 83.7 (C-18), 67.2 (C-17), 67.0 (C-19), 38.1 (C-16), and 18.3 (C-20)] and one methoxy group [δ_H_ 3.70 (3H, s, 15-OCH_3_); δ_C_ 50.5 (15-OCH_3_)] (Table 1). These chemical shift patterns for **1** suggest that D-olivose (C) has been hydrolyzed to generate a linear structure containing the methoxy group; this was verified by COSY correlations from H-16 to H-20 and HMBC correlations involving 15-OCH_3_ (δ_H_ 3.70) and H-16 (δ_H_ 2.77/2.63) with C-15 (δ_C_ 172.7) (Figure 1D). This theory was also confirmed by comparisons of the chemical shift values in the linear structure with D-olivose (C) of everninimicin H [12,13]. On the basis of the HMBC analysis, the correlation of H-18 (δ_H_ 3.52) with C-9 (δ_C_ 99.8) was identified, indicating that the linear structure was connected at C-9 (Figure 1D). A protonated molecular ion at *m*/*z* 179.0907 [M + H]^+^ (calculated for C_7_H_15_O_5_^+^, 179.0914, error = 3.9 ppm) was identified from the LC-MS/MS fragmentation analysis and supported the aforementioned structure elucidation (Figure S5, Supporting Information).

Sporacin B (**2**) was isolated as a pale yellowish gum, and its molecular formula was determined as C_29_H_41_Cl_2_NO_15_ based on a sodiated molecular ion at *m*/*z* 736.1726 [M + Na]^+^ in the HRESIMS (calculated for C_29_H_41_Cl_2_NNaO_15_, 736.1751, error = 3.4 ppm) and an ammonium adduct ion at *m*/*z* 731.2173 [M + NH_4_]^+^ in the HRESIMS (calculated for C_29_H_45_Cl_2_N_2_O_15_, 731.2197, error = 3.3 ppm). The ^1^H NMR and HSQC analyses of **2** were quite similar to those of **1**, with the principal difference being the absence of the methoxy group signal in **2**. In addition, significant downfield-shifted proton and carbon chemical signals were observed in the linear structure of **2**. The methylene group signal at H-16a showed a 0.20 ppm downfield shift, and the signal putative representing H-16b showed a 0.11 ppm upfield shift relative to the same residue in compound **1**, respectively (Figure 1E). In addition, the most obvious differences were confirmed; the carbon chemical shift for C-17 was downfield-shifted (δ_C_ 69.0 in **2**; δ_C_ 67.2 in **1**), and that of C-18 was upfield-shifted (δ_C_ 71.8 in **2**; δ_C_ 83.7 in **1**) (Figure 1E). These major differences suggested that the linear D-olivose moiety was to be connected to C-17 in **2** rather than C-18 in **1**. This assignment was further verified based on the HMBC correlation of H-17 (δ_H_ 4.28) with C-9 (δ_C_ 101.5) (Figure 1D). Furthermore, key LC-MS/MS fragmentation analyses led to identification of the linear D-olivose moiety in **2** based on a sodiated molecular ion at *m*/*z* 187.0571 [M + Na]^+^ (calculated for C_6_H_12_NaO_5_, 187.0582, error = 3.7 ppm) (Figure S11, Supporting Information).

The chemical structures of compounds **1**–**3** were fully assigned by 1D and 2D NMR spectroscopic and mass spectrometry analysis. Despite being a known entity, the full assignments of NMR data for **3** are now, for the first time, reported in this study (Figures S16–S21 and Table S1, Supporting Information).

The isolated compounds **1**–**3** were evaluated for their antibacterial activity against MRSA. As shown in Table 2, compounds **1** and **2** exhibited weak antibacterial activities, with values of 47.7 and 31.3 µM, respectively. This result indicated that the connectivity between olivose (B) and acyclic olivose (C) plays an important role in increasing the antibacterial activity against MRSA. On the other hand, compound **3** did not show any activity against MRSA. Through a comparison of compound **3** with previously reported everninomicins, we inferred that the linear structure of the olivose (C) in **3** had a negative effect on the antibacterial activity against MRSA.

## 3. Materials and Methods

### 3.1. General Experimental Procedures

Optical rotations were measured on a Perkin-Elmer 241 Polarimeter (PerkinElmer, Inc., Waltham, MA, USA). Infrared (IR) was recorded on a Nicolet 6700 FT-IR spectrometer (Thermo Fisher Scientific, Waltham, MA, USA). The NMR spectra (^1^H, COSY, HSQC, and HMBC) were obtained in CD_3_OD with a Bruker Avance III HD 400 MHz (Billerica, MA, USA) spectrometer. The HRESIMS data were acquired with a Bruker MaXis™ 4G ESI-QTOF (Billerica, MA, USA) mass spectrometer. Fractionation was conducted by the Teledyne ISCO Combiflash RF-200 automated flash chromatography system (Lincoln, NE, USA), equipped with a Teledyne RediSep flash chromatography column containing an isolute ENV+ resin. Semi-preparative high-performance liquid chromatography (HPLC) was carried out using a Shimadzu Prominence HPLC system and a Phenomenex Luna C18 column (250 × 30 mm). UHPLC-HRMS was acquired using a Bruker MaXis™ 4G ESI-QTOF (Billerica, MA, USA) mass spectrometer coupled with a Waters Acquity UPLC system operated by Bruker Hystar software v3.2 and a C18 column (Phenomenex Kinetex 2.6 µm, 2.1 mm × 100 mm). The Bruker timsTOF Pro instrument (Billerica, MA, USA) was used for the trapped ion mobility MS analysis using direct infusion with 0.003 mL/min of flow rate and an ESI+ ionization source. The parameters were as follows: nebulizer gas 0.4 bar, dry gas 3.5 L/min, source temperature 220 °C, and ESI voltage 4200V (+). MS spectra were collected using the following parameters: tims ramp time = 350 ms, PASEF on, scan range (*m*/*z*, 20–1000; 1/k_0_, 0.70–1.00 V·s/cm^2^).

### 3.2. Biological Material

A sample of *Phallusia nigra* was collected on 13 August 2019 from a canal in Big Torch Key (24°42′19.5′′ N, 81°25′54.4′′ W) in Florida. A voucher specimen is deposited at the School of Pharmacy, University of Wisconsin—Madison, Madison, WI, USA. A piece of ascidian (~1 cm^3^) was ground in 1 mL sterile seawater, and 150 µL was plated using a sterile L-shaped spreader onto Gauze 1 medium formulated with 50% artificial seawater. Gauze 1 was supplemented with 50 µg/mL cycloheximide, 25 µg/mL nystatin, and 25 µg/mL nalidixic acid. Plates were incubated at 28 °C, and colonies were isolated over the course of four weeks.

### 3.3. Strain Genome Sequencing, Assembly, and Validation

Next-generation sequencing was performed with PacBio Sequel platforms using two Sequel single-molecule real-time (SMRT) cells (University of Wisconsin, Madison [UW-Madison], Biotechnology Center). PacBio data were corrected, trimmed, and assembled with Canu v1.8 using the parameter “genomeSize = 8 m” on the Center for High Throughput Computing (CHTC) computing resources [14]. BUSCO v5.4.3 and QUAST v5.0.2 were used to assess the genome assembly based on completeness and quality, respectively [15].

### 3.4. Annotation of Biosynthetic Gene Clusters (BGCs)

To identify BGCs related to secondary metabolism, the genome sequence, in the Fasta format, was annotated by antiSMASH v7.0.0 [8]. Biosynthetic gene cluster (BGC) prediction was conducted using antiSMASH v7.0.0 with relaxed detection strictness and the activation of additional analysis modules, including KnownClusterBlast, ClusterBlast, SubClusterBlast, MIBiG cluster comparison, Active Site Finder, RRE Finder, Cluster Pfam analysis, Pfam-based Gene Ontology (GO) term annotation, TIGRFAMs analysis, and transcription factor binding site (TFBS) analysis.

### 3.5. Species Classification and BGC Analysis

The Genome Taxonomy Database (GTDB-tk v2) was used to classify taxonomy for WMMD956 using GTDB release 207 [16,17]. WMMD956 was queried against 29,955 gene cluster families (GCFs) clustered from 1,225,071 BGCs taken from publicly available sources using the BiG-SLiCE software v1.1.1 with a clustering threshold of 900.0 with BiG-FAM [18,19].

### 3.6. Molecular Networking

A molecular network was created using the online workflow at GNPS (https://gnps.ucsd.edu, accessed on 23 March 2023) [20]. The precursor ion mass tolerance was set to 0.02 Da, and the MS/MS fragment ion tolerance was set to 0.02 Da. The molecular networks were produced using a cosine score threshold of 0.7 and a minimum of three matched peaks. The library search was set up using a cosine score threshold of 0.6 and at least three matched peaks. The spectra in the network were searched against all GNPS spectral libraries. The library spectra were filtered in the same manner as the input data. The results were visualized in Cytoscape 3.9.1 (www.cytoscape.org, accessed on 23 March 2023).

### 3.7. Fermentation

WMMD956 was inoculated into 25 × 150 mm culture tubes containing DSC medium (5 g soluble starch, 10 g glucose, 5 g peptone, 5 g yeast extract per liter of 50% artificial seawater) and grown at 28 °C with agitation at 200 rpm until turbid. These seed cultures were then transferred into 1 L of DSC in a fernbach flask, which was incubated at 28 °C for seven days at 200 rpm. Fernbach flasks containing DSC medium (10 × 1 L) with 50 μM iron (III) sulfate and Diaion HP-20 (7% by wet weight) were inoculated with 35 mL from the intermediate culture and shaken at 200 RPM for 14 days at 28 °C.

### 3.8. Extraction and Isolation

Filtered HP-20 resin was washed using water; then, the resin was extracted with acetone. The acetone extract (4.2 g) was divided into five fractions using ENV+ combiflash chromatography, eluting with mixtures of MeOH-H_2_O (water, 25%, 50%, 75%, and 100% MeOH), yielding 1400, 198, 314, 691, and 652 mg of dried eluents, respectively. The dried water fraction (A) was isolated by preparative HPLC (Phenomenex, Onyx Monolithic semi-PREP C18 100 × 10 mm i.d.) with a gradient elution from 10 to 100% aqueous MeCN with 0.1% acetic acid over 20 min (flow rate: 20 mL/min) to afford 20 fractions. These 20 subfractions were combined to seven subfractions (A1–A7) based on the HPLC analysis and biological evaluation. Among them, compounds **1** (*t*_R_ 7.9 min, 1.5 mg) and **2** (*t*_R_ 9.6 min, 0.8 mg) were isolated from subfraction A6 (29.1 mg) using semi-prep HPLC equipped with a Phenomenex Luna 5 *μ* C18(2) column (250 mm × 10 mm i.d., 5 μm; flow rate: 4.0 mL/min for 20 min) with a gradient solvent system of 50–85% MeCN with 0.1% acetic acid. The 75% MeOH fraction (D) was divided into six subfractions (D1–D6) on the basis of the NMR and HPLC analysis and biological evaluation. Compound **3** (*t*_R_ 11.1 min, 1.9 mg) was afforded from subfraction D5 using semi-prep HPLC equipped with a Phenomenex Luna 5 *μ* C18(2) column (250 mm × 10 mm i.d., 5 μm; flow rate: 4.0 mL/min for 20 min) with a gradient solvent system of 55–60% MeCN with 0.1% acetic acid.

Sporacin A (**1**): Pale brownish gum; ${[\alpha ]\;\;}_{D}^{25}$ –45.0 (*c* 0.002, MeOH); IR (KBr) *v*_max_ 3394, 2934, 1731, 1545, 1456, 1395, 1348, 1251, 1177, 1128, 1105, 1068, 1036, 1002, 952, 925 cm^−1^; UV (MeOH) λ_max_ 209 nm; ^1^H MNR (400 MHz) and ^13^C NMR (100 MHz) data in methanol-*d*_4_; see Table 1; HRESIMS (positive-ion mode) *m*/*z* 750.1875 [M + Na]^+^ (calcd for C_30_H_43_Cl_2_NNaO_15_, 750.1907, error = 4.3 ppm) and *m*/*z* 745.2321 [M + NH_4_]^+^ (calcd for C_30_H_47_Cl_2_N_2_O_15_, 745.2353, error = 4.3 ppm).

Sporacin B (**2**): Pale brownish gum; ${[\alpha ]\;\;}_{D}^{25}$ –56.2 (*c* 0.001, MeOH); IR (KBr) *v*_max_ 3391, 2927, 2853, 1736, 1661, 1617, 1558, 1456, 1382, 1246, 1108, 1029 cm^−1^; UV (MeOH) λ_max_ 213 nm; ^1^H MNR (400 MHz) and ^13^C NMR (100 MHz) data in methanol-*d*_4_; see Table 1; HRESIMS (positive-ion mode) *m*/*z* 736.1730 [M + Na]^+^ (calcd for C_29_H_41_Cl_2_NNaO_15_, 736.1751, error = 2.9 ppm) and *m*/*z* 731.2173 [M + NH_4_]^+^ (calcd for C_29_H_45_Cl_2_N_2_O_15_, 731.2197, error = 3.3 ppm).

### 3.9. Screening for Inhibitors of MRSA Growth

Assay plates for antimicrobial testing were made ahead of time using the Eco 650 acoustic liquid handler. Overall, 500, 250, 125, 62.5, 32.5, 15, 7.5, 5, 2.5, 0.4, 0.25, 0.125, 0.05, 0.025, and 0.01 nL of compounds was transferred to each quadrant of a clear 384-well plate. The following control was used for MRSA328 (Vancomycin, 1 μg/mL). A single colony of MRSA328 was picked from a solid agar plate into 5 mL of a liquid culture and grown for 18 h shaking at 37 °C. This O/N culture was diluted to 0.5 McFarland units, and this stock was further diluted 1:300 for use in HTS assays. A 50 μL aliquot per well of the diluted culture was added to each well of the 384 well assay plate using the Multidrop instrument. Microorganisms were incubated with the compound O/N at 37 °C. Microorganism growth was measured by collecting an end point absorbance reading at OD600 using a BMG CLARIOStar plate reader. The collected data were uploaded to CDD within 24–48 h of the read time, and the % inhibition was calculated based on the OD values of DMSO-only wells.

## 4. Conclusions

Marine-derived *Micromonospora* spp. bacteria are considered the largest reservoir of microbial natural sources, producing an abundance of biologically active secondary metabolites and representing a broad range of chemical scaffolds. Metabolomics and genomics approaches, especially *hcapca* and molecular networking (GNPS), as well as antiSMASH, were verified as effective and powerful strategies by which to prioritize novel strains, thus enhancing natural product discovery potentials. With these approaches, WMMD956 was identified as a highly novel strain likely producing unique chemical diversity, enabling the discovery **1** and **2**.

## Figures and Tables

**Table 1:** ^1^H (400 MHz) and ^13^C (100 MHz) NMR Spectroscopic Data for **1** and **2** in methanol-*d*_4_ (*δ* in ppm, *J* values in Hz).

Position	1		2	
	*δ*_C_	*δ*_H_ (*J* in Hz)	*δ*_C_	*δ*_H_ (*J* in Hz)
1	114.2		114.2	
2	153.7		154.0	
3	116.4		116.1	
4	n.d. *^a^*		n.d. *^a^*	
5	119.2		119.0	
6	134.4		134.3	
7	166.2		166.4	
8	17.0	2.38, s	18.2	2.37, s
2-OCH_3_	61.1	3.91, s	62.2	3.90, s
9	99.8	4.71, d (9.3)	101.5	4.81, d (9.0)
10a	35.8	2.45, m	37.2	2.45, m
10b		1.61, m		1.59, m
11	72.4	3.99, m	73.6	3.99, m
12	76.2	4.80, t (9.4)	77.2	4.79, m
13	70.6	3.60, m	72.2	3.69, m
14	17.3	1.35, d (6.0)	18.5	1.38, d (6.2)
15	172.7			
16a	38.1	2.77, dd (16.3, 4.9)	36.8	2.97, dd (16.6, 4.9)
16b		2.63, dd (16.2, 8.0)		2.52, dd (16.6, 4.7)
17	67.2	4.33, m	69.0	4.28, m
18	83.7	3.52, m	71.8	3.58, m
19	67.0	3.95, m	68.4	4.31, m
20	18.3	1.26, d (6.2)	18.8	1.45, d (6.4)
15-OCH_3_	50.5	3.70, s		
21	92.8	5.03, d (4.4)	94.1	5.02, d (4.3)
22a	40.1	2.43, d (13.7)	41.4	2.44, d (13.8)
22b		2.08, d (13.6)		2.06, d (13.6)
23	89.8		89.8	
24	84.4	3.62, m	84.3	3.59, m
25	65.9	3.50, m	67.1	3.49, m
26	16.4	0.77, d (6.0)	17.7	0.76, d (6.1)
27	18.4	1.69, s	19.6	1.67, s
24-OCH_3_	54.7	3.34, s	56.0	3.35, s

*^a^* n.d.: not detected.

**Table 2:** Antibacterial activity evaluation for the compounds **1**–**3** against MRSA.

Compound	MRSA
	IC_50_ (µM)
**1**	47.7
**2**	31.3
**3**	>100

## Data Availability

The original data presented in the study are included in the arti cle/Supplementary Material; further inquiries can be directed to the corresponding author.

## Supplementary Materials

The following Supporting Information can be downloaded at https://www.mdpi.com/article/10.3390/md23080316/s1: Figure S1. Comprehensive antiSMASH results for WMMD956. Figure S2. Pictures of the disk diffusion assay on the fractions A and D and acetone extract. Figure S3. Molecular ion networking analysis of crude extract of *Micromonospora* sp. WMMD956. Figure S4. HRESIMS spectrum of **1**. Figure S5. MSMS fragmentation of the linear structure (C) ion *m*/*z* 179.0907 [M + H]^+^ (calc for C_7_H_15_O_5_^+^, 179.0914, error = 3.9 ppm) in **1**. Figure S6. ^1^H NMR spectrum of **1** in methanol-*d_4_*. Figure S7. COSY spectrum of **1** in methanol-*d_4_*. Figure S8. HSQC spectrum of **1** in methanol-*d_4_*. Figure S9. HMBC spectrum of **1** in methanol-*d_4_*. Figure S10. HRESIMS spectrum of **2**. Figure S11. MSMS fragmentation of the linear structure (C) ion *m*/*z* 187.0575 [M + Na]^+^ (calc for C_6_H_12_NaO_5_^+^, 187.0582, error = 3.7 ppm) in **2**. Figure S12. ^1^H NMR spectrum of **2** in methanol-*d_4_*. Figure S13. COSY spectrum of **2** in methanol-*d_4_*. Figure S14. HSQC spectrum of **2** in methanol-*d_4_*. Figure S15. HMBC spectrum of **2** in methanol-*d_4_*. Figure S16. HRESIMS spectrum of **3**. Figure S17. ^1^H NMR spectrum of **3** in methanol-*d_4_*. Figure S18. COSY spectrum of **3** in methanol-*d_4_*. Figure S19. HSQC spectrum of **3** in methanol-*d_4_*. Figure S20. HMBC spectrum of **3** in methanol-*d_4_*. Figure S21. TOCSY spectrum of **3** in methanol-*d_4_*. Table S1. ^1^H (400 MHz) and ^13^C (100 MHz) NMR Spectroscopic Data for compound **3** in methanol-*d*_4_ (*δ* in ppm; *J* values in Hz).
