# Supplementary material for: Discovery of New Everninomicin Analogs from a Marine-Derived Micromonospora sp. by Metabolomics and Genomics Approaches
Source: Mar Drugs. 2025 Jul 31;23(8):316. doi: 10.3390/md23080316 (PMC12387530; doi:10.3390/md23080316)
Supplement: Supplementary file 1 [file marinedrugs-23-00316-s001.zip › WMMD956_Everninomycin analogs_Marine Drugs_SI (THL).pdf]

Supporting Information for

Discovery of new everninomicin analogs from a marine-derived *Micromonospora* sp. by metabolomics and genomics approaches

Tae Hyun Lee [1], Nathan J. Brittin [1], Imraan Alas [1], Christopher D. Roberts [1], Shaurya Chanana [1], Doug R. Braun [1], Spencer S. Ericksen [2], Song Guo [2], Scott R. Rajski [1], and Tim S. Bugni [1,2,3]*

[1] Pharmaceutical Sciences Division, University of Wisconsin–Madison, Madison, Wisconsin 53705, United States; taehyun.lee@wisc.edu (T.H.L); nbrittin@wisc.edu (N.J.B); alas@wisc.edu (I.A.); cdroberts3@wisc.edu (C.D.R.); schanana@wisc.edu (S.C.); drbraun1@wisc.edu (D.R.B.); scott.rajski@wisc.edu (S.R.R.); tim.bugni@wisc.edu (T.S.B.)
[2] Small Molecule Screening Facility, UW Carbone Cancer Center, Madison, Wisconsin, United States; ssericksen@wisc.edu (S.S.E.); sguo6@wisc.edu (S.G.); tim.bugni@wisc.edu (T.S.B.)
[3] Lachman Institute for Pharmaceutical Development, University of Wisconsin–Madison, Madison, Wisconsin, United States; tim.bugni@wisc.edu (T.S.B.)
*Correspondence: tim.bugni@wisc.edu

Contents **Page #**

**Figure S1**. Comprehensive antiSMASH results for WMMD956.	S3
**Figure S2**. Pictures of the disk diffusion assay on the fractions A and D and acetone extract.	S4
**Figure S3**. Molecular ion networking analysis of crude extract of *Micromonospora* sp. WMMD956.	S5–S6
**Figure S4**. HRESIMS spectrum of **1**.	S7
**Figure S5**. MSMS fragmentation of the linear structure (C) ion *m/z* 179.0907 $[M + H]^+$ (calc for $C_7H_{15}O_5^+$, 179.0914, error = 3.9 ppm) in **1**.	S8
**Figure S6**. ^1H NMR spectrum of **1** in methanol-d_4.	S9
**Figure S7**. COSY spectrum of **1** in methanol-d_4.	S10
**Figure S8**. HSQC spectrum of **1** in methanol-d_4.	S11
**Figure S9**. HMBC spectrum of **1** in methanol-d_4.	S12
**Figure S10**. HRESIMS spectrum of **2**.	S13
**Figure S11**. MSMS fragmentation of the linear structure (C) ion *m/z* 187.0575 $[M + Na]^+$ (calc for $C_6H_{12}NaO_5^+$, 187.0582, error = 3.7 ppm) in **2**.	S14
**Figure S12**. ^1H NMR spectrum of **2** in methanol-d_4.	S15
**Figure S13**. COSY spectrum of **2** in methanol-d_4.	S16
**Figure S14**. HSQC spectrum of **2** in methanol-d_4.	S17
**Figure S15**. HMBC spectrum of **2** in methanol-d_4.	S18
**Figure S16**. HRESIMS spectrum of **3**.	S19
**Figure S17**. ^1H NMR spectrum of **3** in methanol-d_4.	S20
**Figure S18**. COSY spectrum of **3** in methanol-d_4.	S21
**Figure S19**. HSQC spectrum of **3** in methanol-d_4.	S22
**Figure S20**. HMBC spectrum of **3** in methanol-d_4.	S23
**Figure S21**. TOCSY spectrum of **3** in methanol-d_4.	S24
**Table S1.** ^1H (400 MHz) and ^{13}C (100 MHz) NMR Spectroscopic Data for Compound 3 in Methanol-d_4 (δ in ppm, J values in Hz).	S25–S26

Figure S1. Comprehensive antiSMASH results for WMMD956.

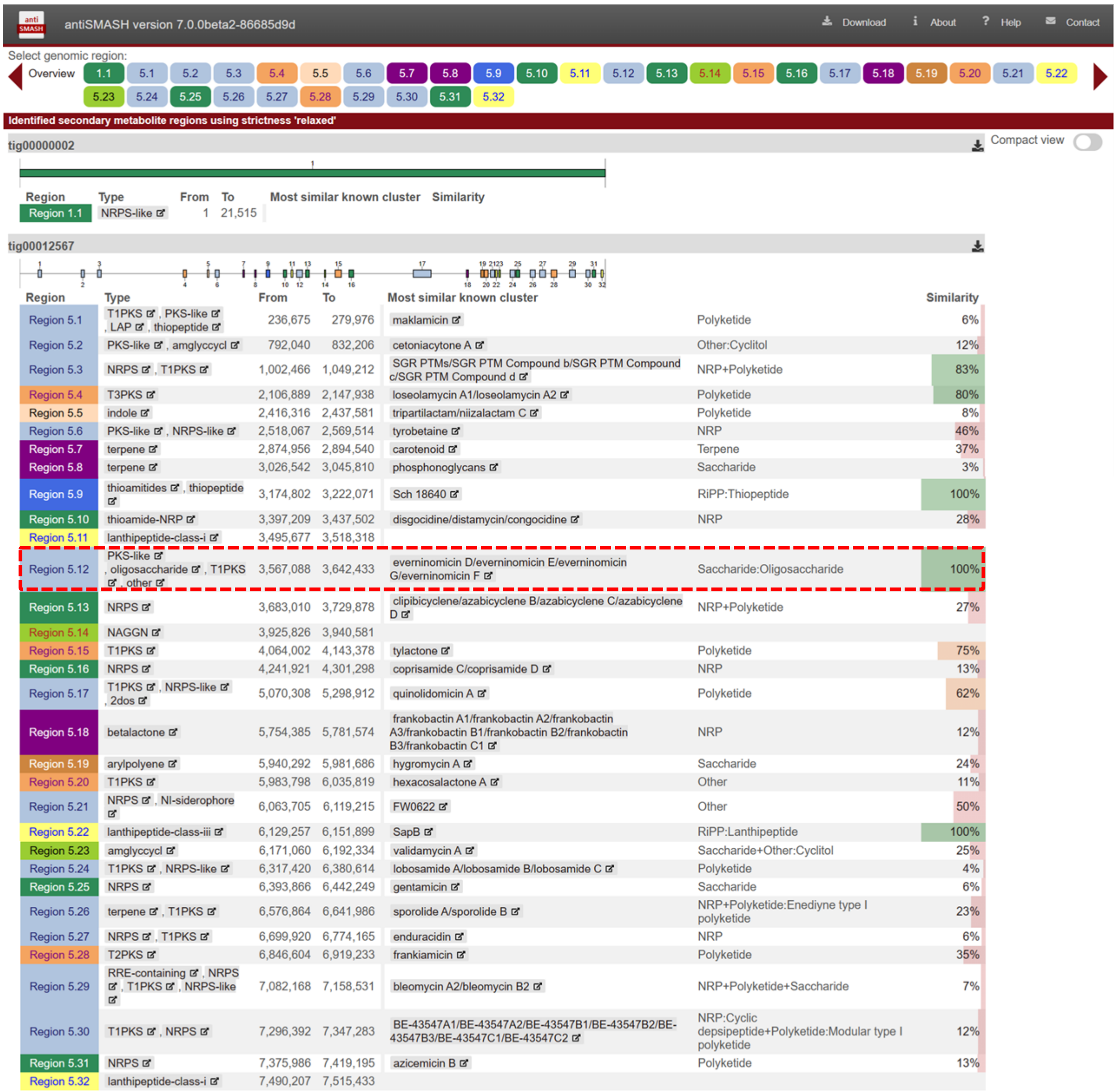

antiSMASH version 7.0.0beta2-86685d9d Download About Help Contact

Select genomic region:

Overview 1.1 5.1 5.2 5.3 5.4 5.5 5.6 5.7 5.8 5.9 5.10 5.11 5.12 5.13 5.14 5.15 5.16 5.17 5.18 5.19 5.20 5.21 5.22 5.23 5.24 5.25 5.26 5.27 5.28 5.29 5.30 5.31 5.32

Identified secondary metabolite regions using strictness 'relaxed'

tig00000002 Compact view

Region	Type	From	To	Most similar known cluster	Similarity
Region 1.1	NRPS-like	1	21,515		

tig00012567

Region	Type	From	To	Most similar known cluster		Similarity
Region 5.1	T1PKS, PKS-like, LAP, thiopeptide	236,675	279,976	maklamicin	Polyketide	6%
Region 5.2	PKS-like, amglyccycl	792,040	832,206	cetoniacytone A	Other:Cyclitol	12%
Region 5.3	NRPS, T1PKS	1,002,466	1,049,212	SGR PTMs/SGR PTM Compound b/SGR PTM Compound c/SGR PTM Compound d	NRP+Polyketide	83%
Region 5.4	T3PKS	2,106,889	2,147,938	loseolamycin A1/loseolamycin A2	Polyketide	80%
Region 5.5	indole	2,416,316	2,437,581	tripartilactam/niizalactam C	Polyketide	8%
Region 5.6	PKS-like, NRPS-like	2,518,067	2,569,514	tyrobetaine	NRP	46%
Region 5.7	terpene	2,874,956	2,894,540	carotenoid	Terpene	37%
Region 5.8	terpene	3,026,542	3,045,810	phosphonoglycans	Saccharide	3%
Region 5.9	thioamitides, thiopeptide	3,174,802	3,222,071	Sch 18640	RiPP:Thiopeptide	100%
Region 5.10	thioamide-NRP	3,397,209	3,437,502	disgocidine/distamycin/congocidine	NRP	28%
Region 5.11	lanthipeptide-class-i	3,495,677	3,518,318			
Region 5.12	PKS-like, oligosaccharide, T1PKS, other	3,567,088	3,642,433	everninomicin D/everninomicin E/everninomicin G/everninomicin F	Saccharide:Oligosaccharide	100%
Region 5.13	NRPS	3,683,010	3,729,878	clipibicyclene/azabicyclene B/azabicyclene C/azabicyclene D	NRP+Polyketide	27%
Region 5.14	NAGGN	3,925,826	3,940,581			
Region 5.15	T1PKS	4,064,002	4,143,378	tylactone	Polyketide	75%
Region 5.16	NRPS	4,241,921	4,301,298	coprisamide C/coprisamide D	NRP	13%
Region 5.17	T1PKS, NRPS-like, 2dos	5,070,308	5,298,912	quinolidomicin A	Polyketide	62%
Region 5.18	betalactone	5,754,385	5,781,574	frankobactin A1/frankobactin A2/frankobactin A3/frankobactin B1/frankobactin B2/frankobactin B3/frankobactin C1	NRP	12%
Region 5.19	arylpolyene	5,940,292	5,981,686	hygromycin A	Saccharide	24%
Region 5.20	T1PKS	5,983,798	6,035,819	hexacosalactone A	Other	11%
Region 5.21	NRPS, NI-siderophore	6,063,705	6,119,215	FW0622	Other	50%
Region 5.22	lanthipeptide-class-iii	6,129,257	6,151,899	SapB	RiPP:Lanthipeptide	100%
Region 5.23	amglyccycl	6,171,060	6,192,334	validamycin A	Saccharide+Other:Cyclitol	25%
Region 5.24	T1PKS, NRPS-like	6,317,420	6,380,614	lobosamide A/lobosamide B/lobosamide C	Polyketide	4%
Region 5.25	NRPS	6,393,866	6,442,249	gentamicin	Saccharide	6%
Region 5.26	terpene, T1PKS	6,576,864	6,641,986	sporolide A/sporolide B	NRP+Polyketide:Enediyne type I polyketide	23%
Region 5.27	NRPS, T1PKS	6,699,920	6,774,165	enduracidin	NRP	6%
Region 5.28	T2PKS	6,846,604	6,919,233	frankiamicin	Polyketide	35%
Region 5.29	RRE-containing, NRPS, T1PKS, NRPS-like	7,082,168	7,158,531	bleomycin A2/bleomycin B2	NRP+Polyketide+Saccharide	7%
Region 5.30	T1PKS, NRPS	7,296,392	7,347,283	BE-43547A1/BE-43547A2/BE-43547B1/BE-43547B2/BE-43547B3/BE-43547C1/BE-43547C2	NRP:Cyclic depsipeptide+Polyketide:Modular type I polyketide	12%
Region 5.31	NRPS	7,375,986	7,419,195	azicemicin B	Polyketide	13%
Region 5.32	lanthipeptide-class-i	7,490,207	7,515,433			

Figure S2. Pictures of the disk diffusion assay on the fractions A and D and acetone extract.

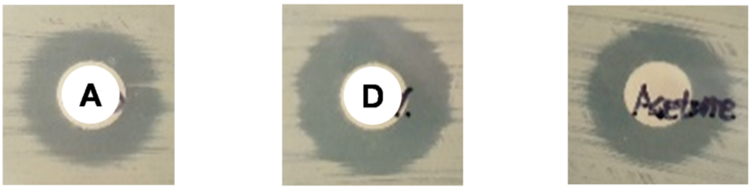

Fraction A **Fraction D** **Acetone ext.**

Figure S3. Molecular ion networking analysis of crude extract of *Micromonospora* sp. WMMD956.

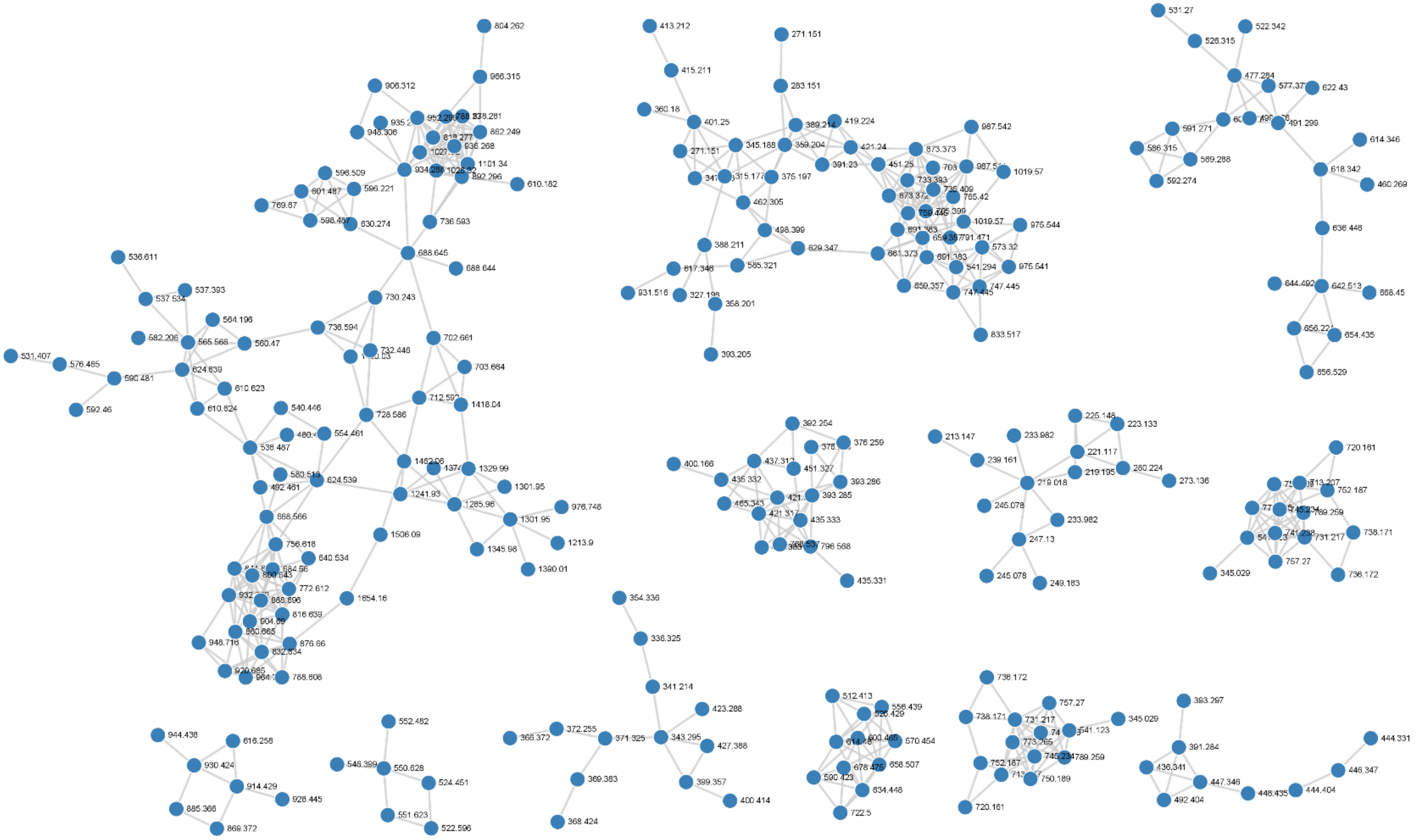

Figure S3. (Continued)

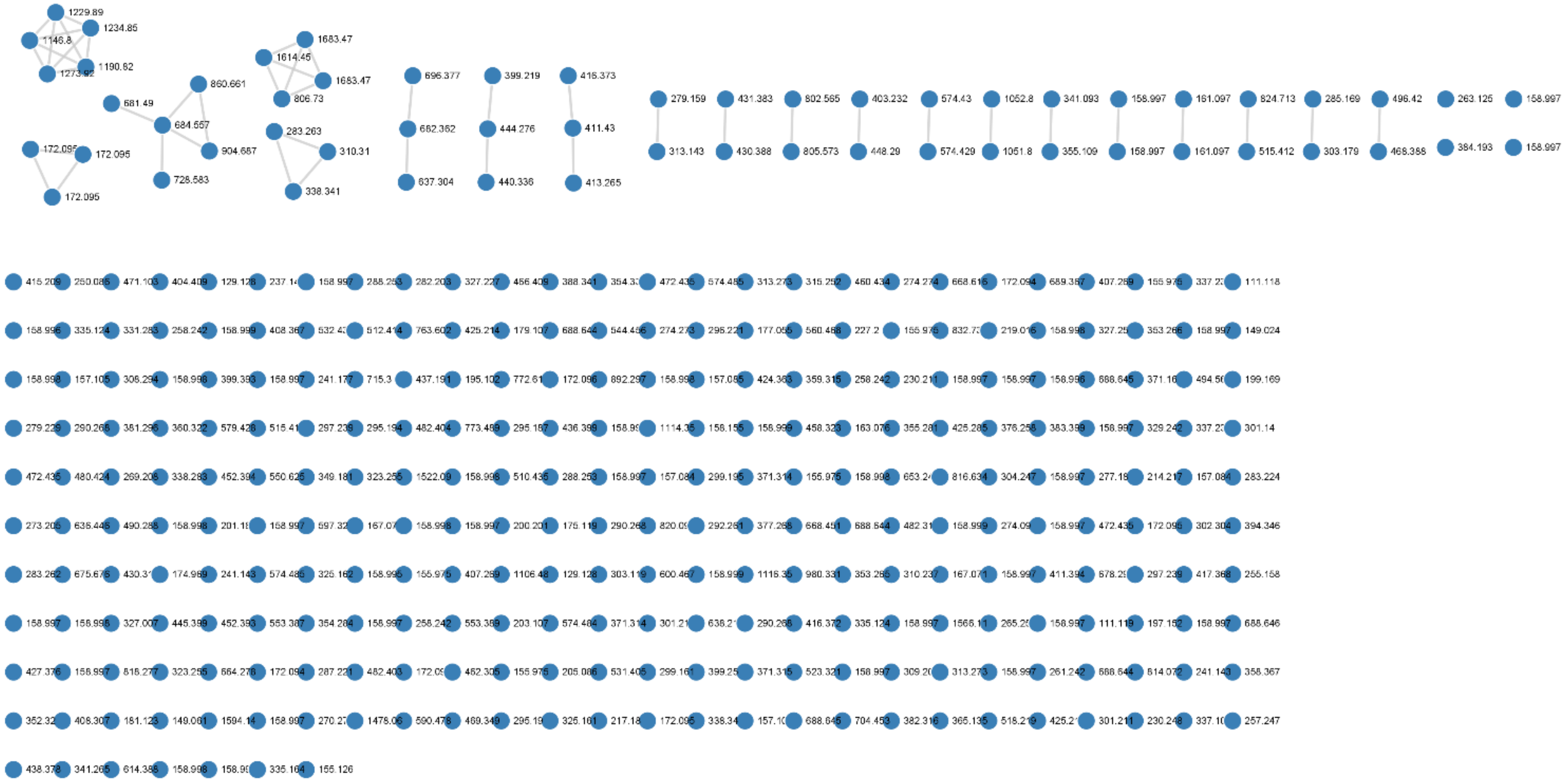

Figure S4. HRESIMS spectrum of **1**.

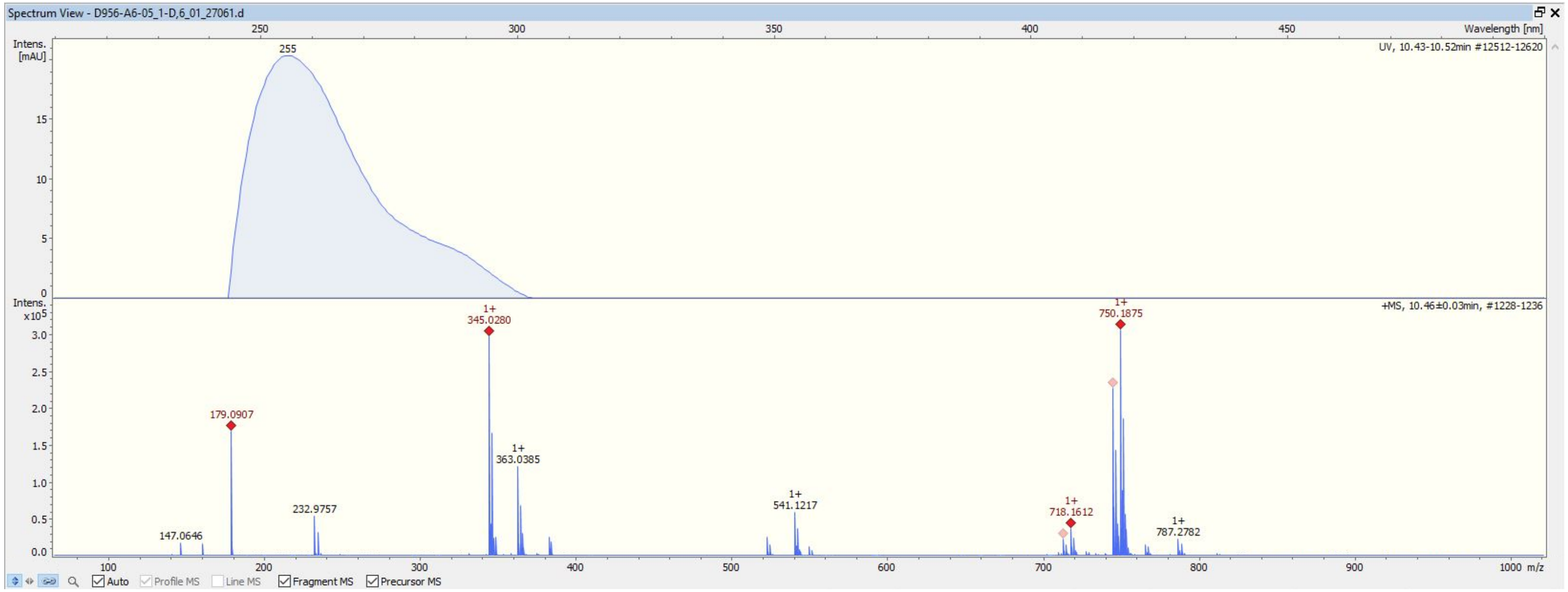

Figure S5. MSMS fragmentation of the linear structure (C) ion *m/z* 179.0907 $[M + H]^+$ (calc for $C_7H_{15}O_5^+$, 179.0914, error = 3.9 ppm) in **1**.

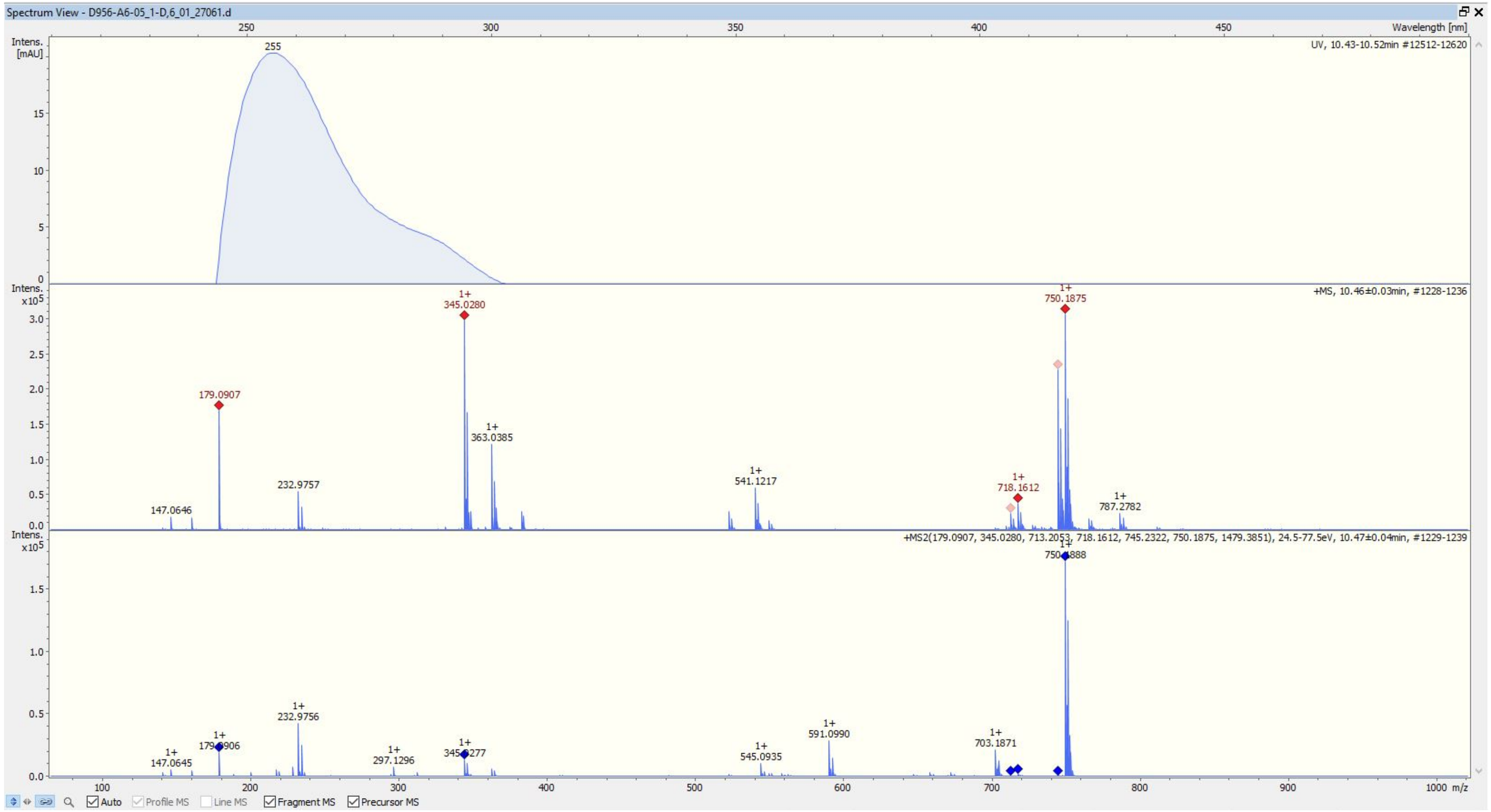

Figure S6. ^{1}H NMR spectrum of **1** in methanol-d_4.

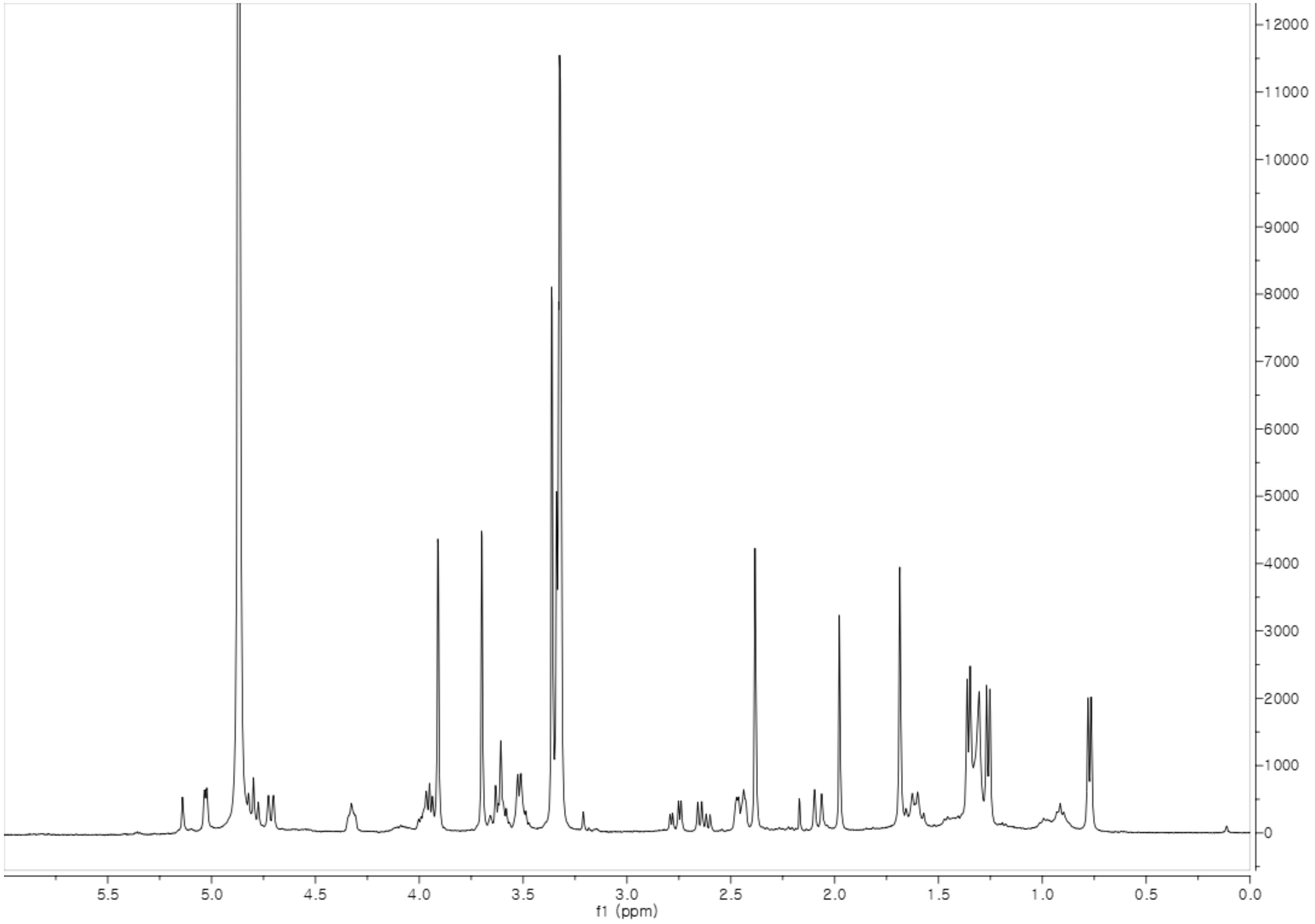

Figure S7. COSY spectrum of **1** in methanol-d_4.

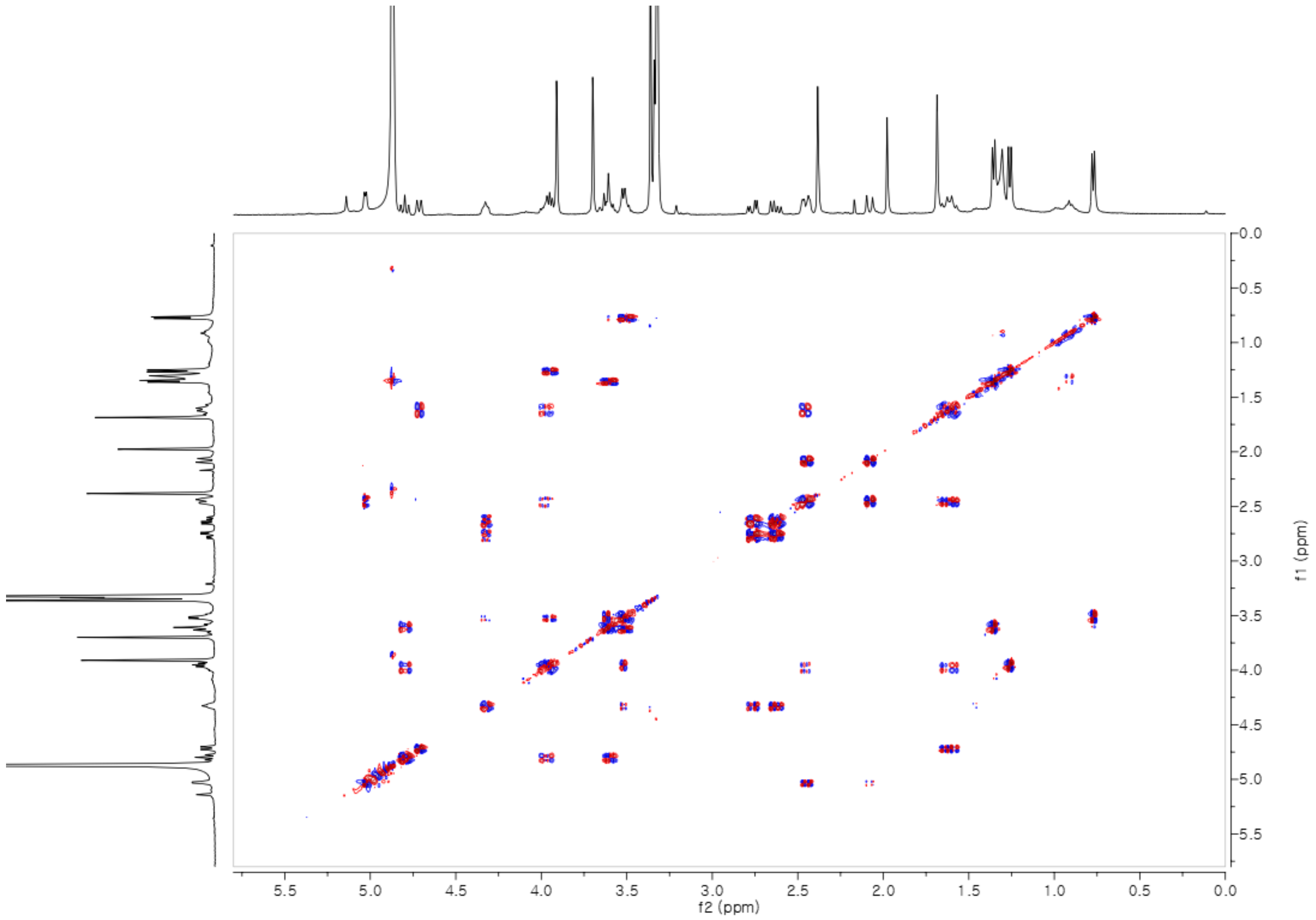

Figure S8. HSQC spectrum of **1** in methanol-d_4.

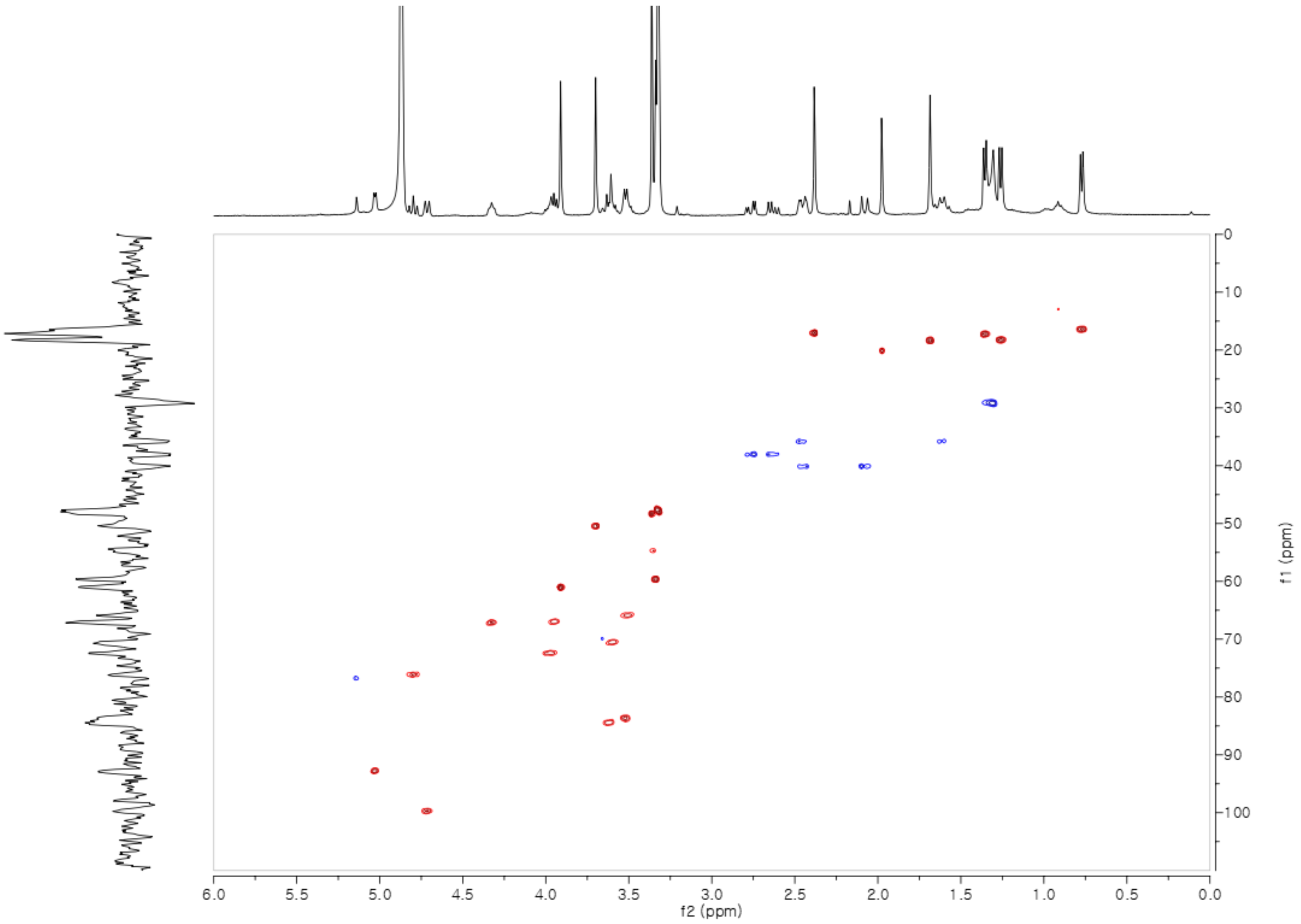

Figure S9. HMBC spectrum of **1** in methanol-d_4.

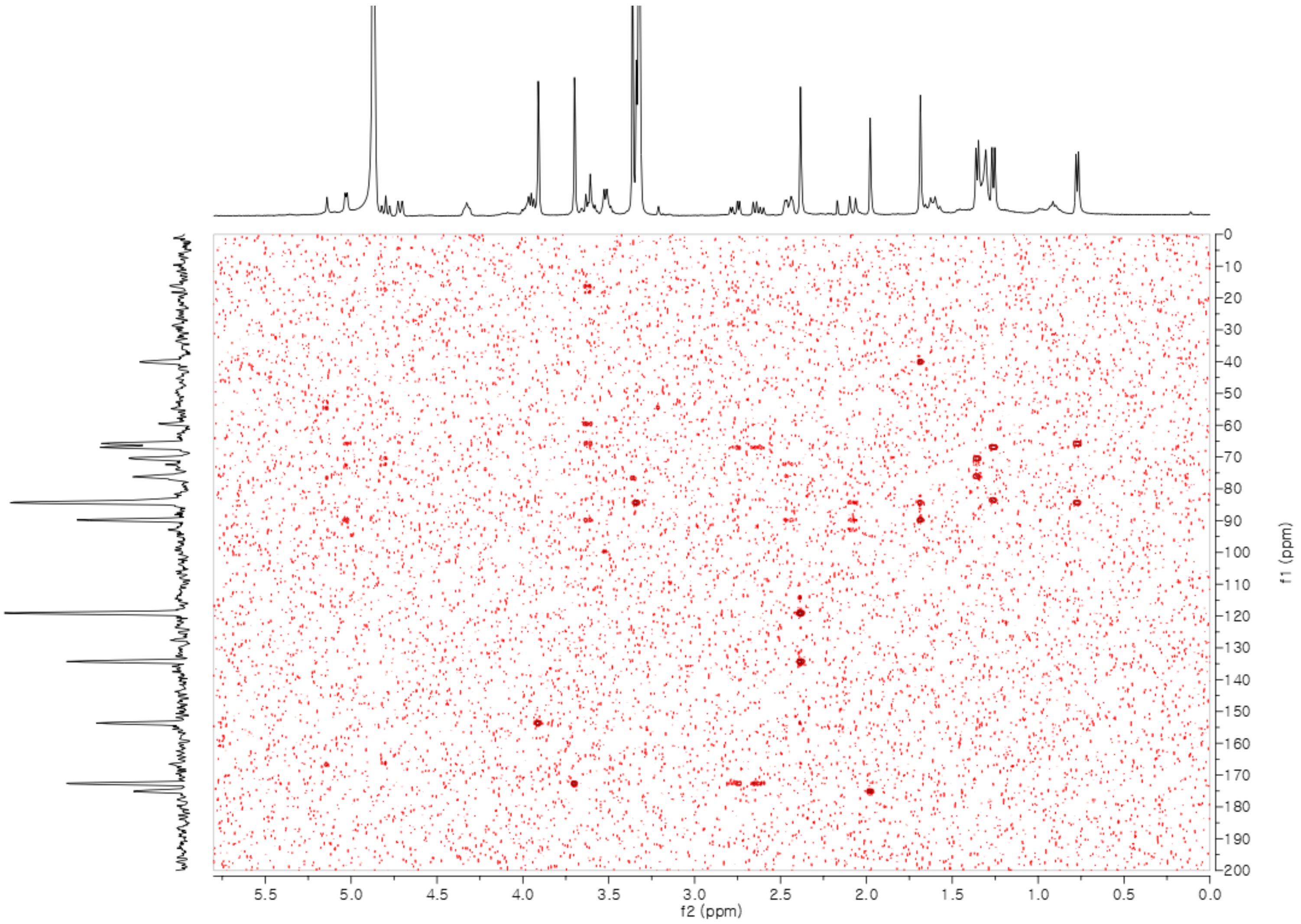

Figure S10. HRESIMS spectrum of **2**.

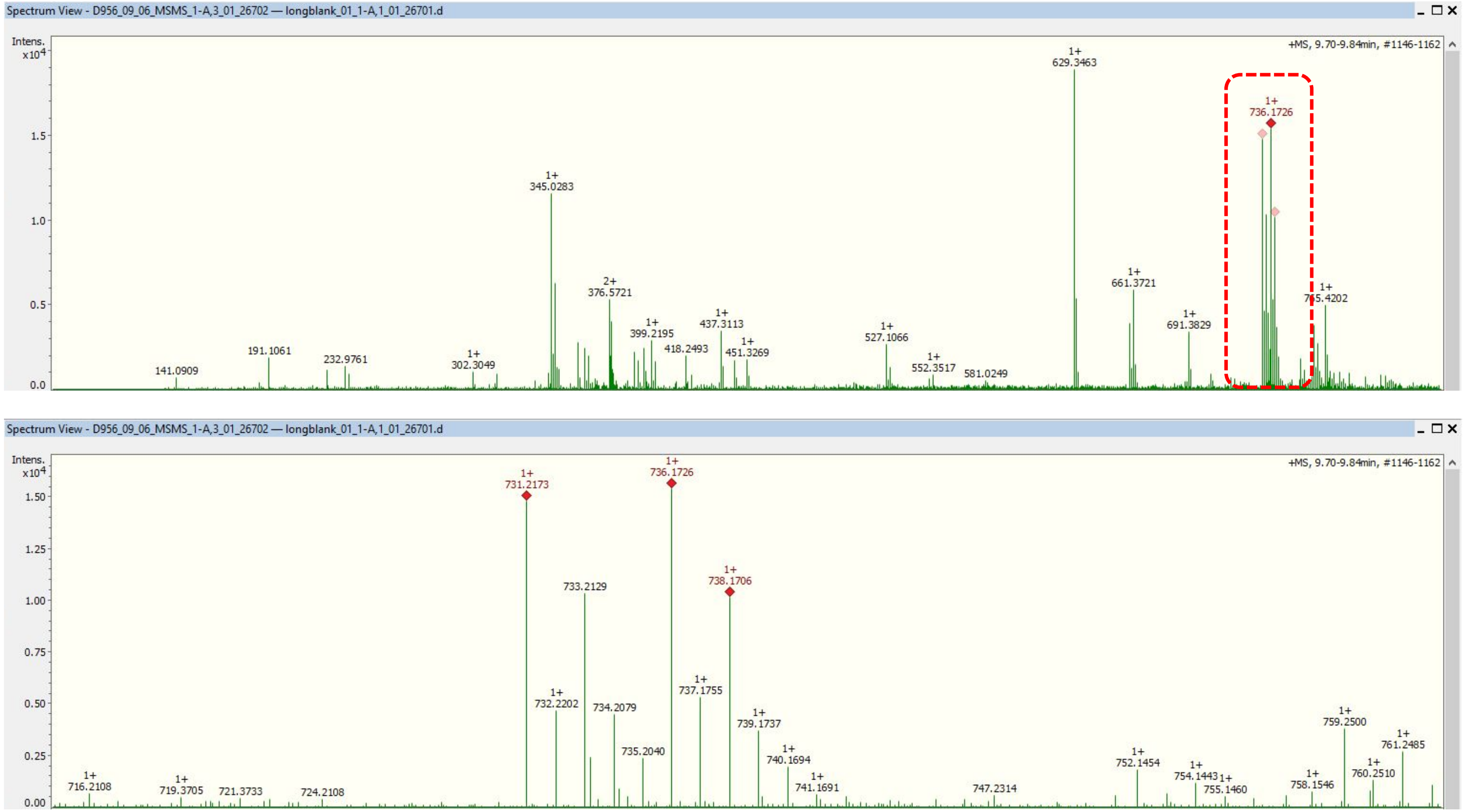

Figure S11. MSMS fragmentation of the linear structure (C) ion *m/z* 187.0575 $[M + Na]^+$ (calc for $C_6H_{12}NaO_5^+$, 187.0582, error = 3.7 ppm) in **2**.

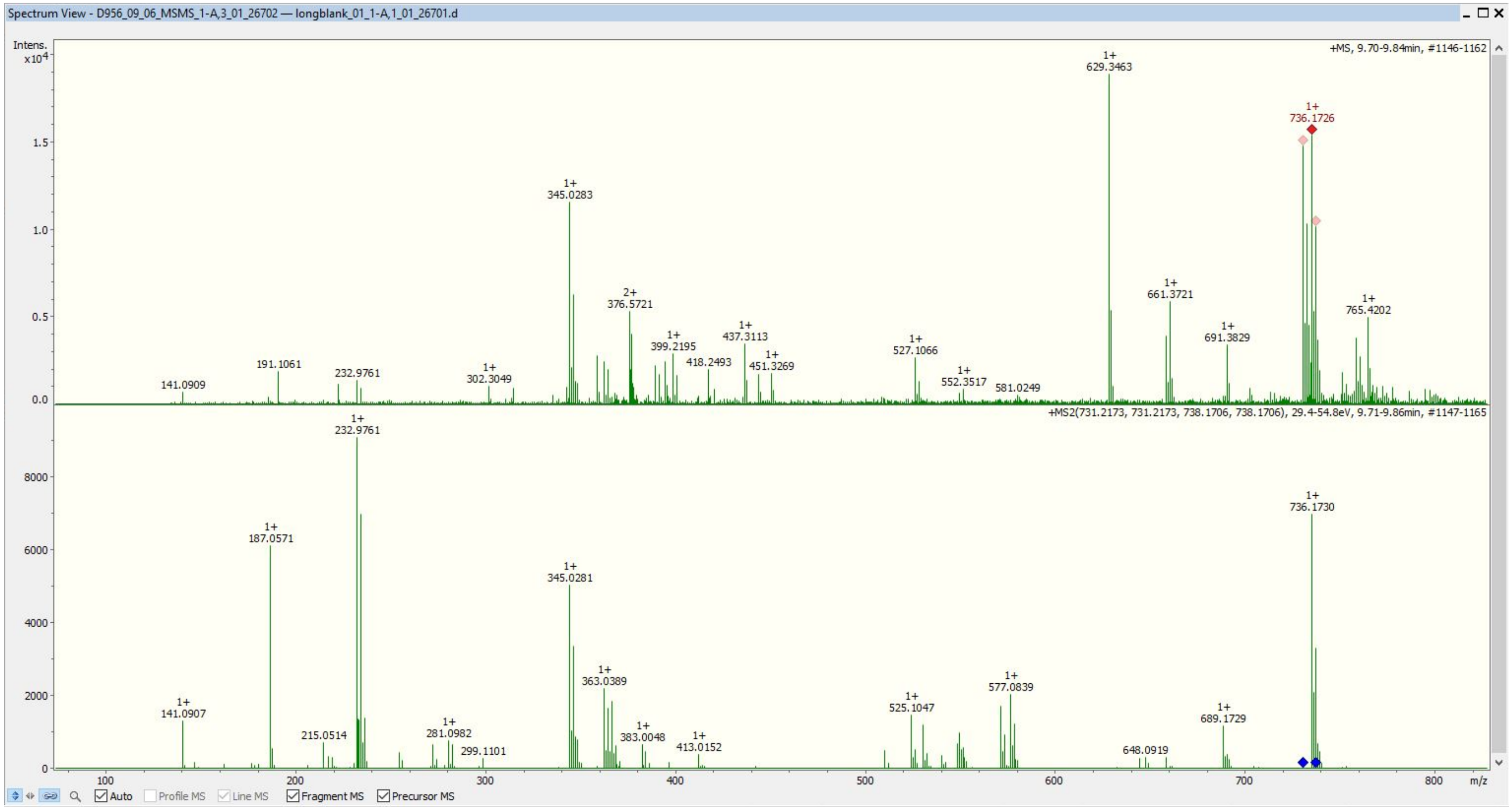

Figure S12. ^{1}H NMR spectrum of **2** in methanol-*d*$_4$.

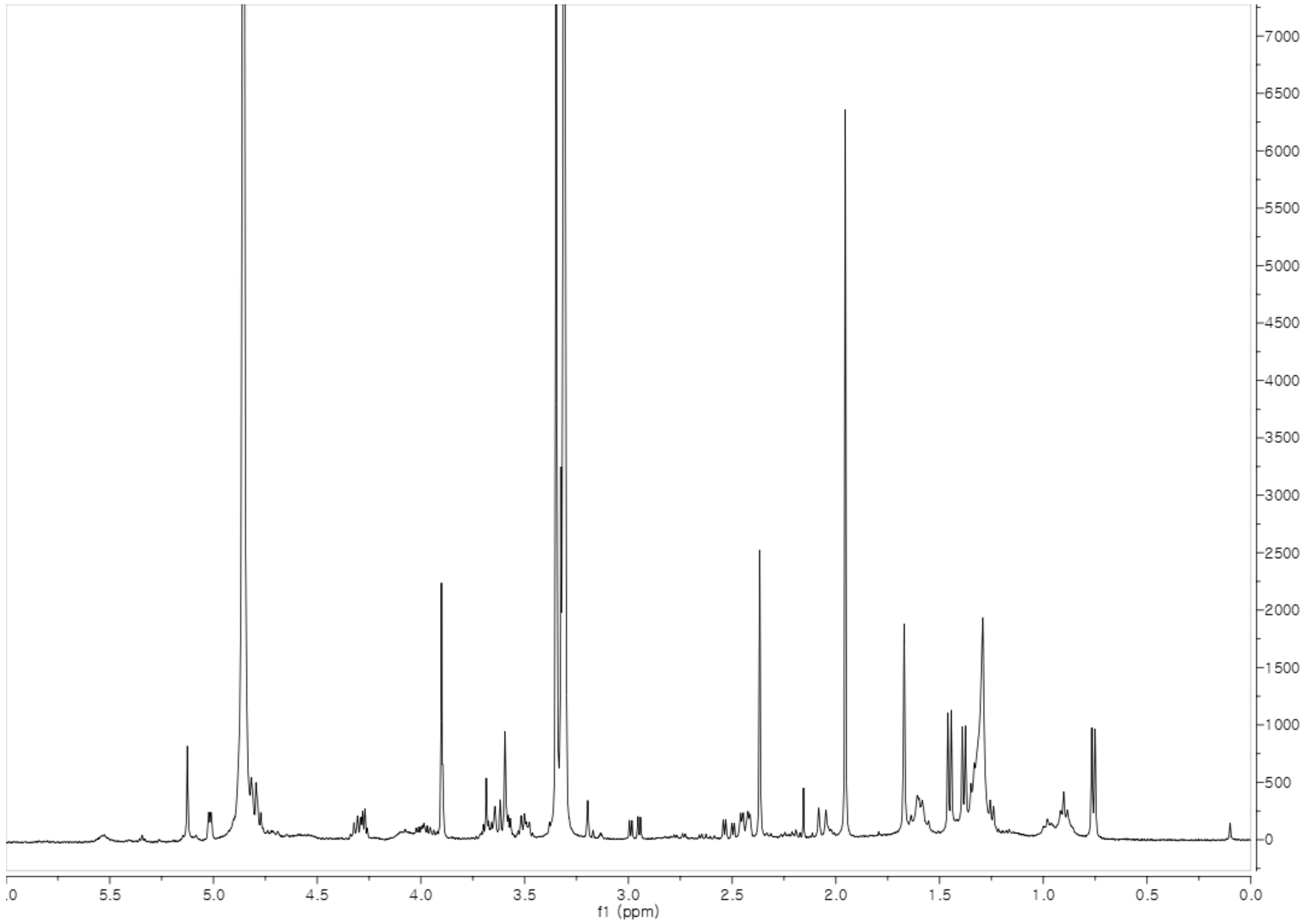

Figure S13. COSY spectrum of **2** in methanol-d_4.

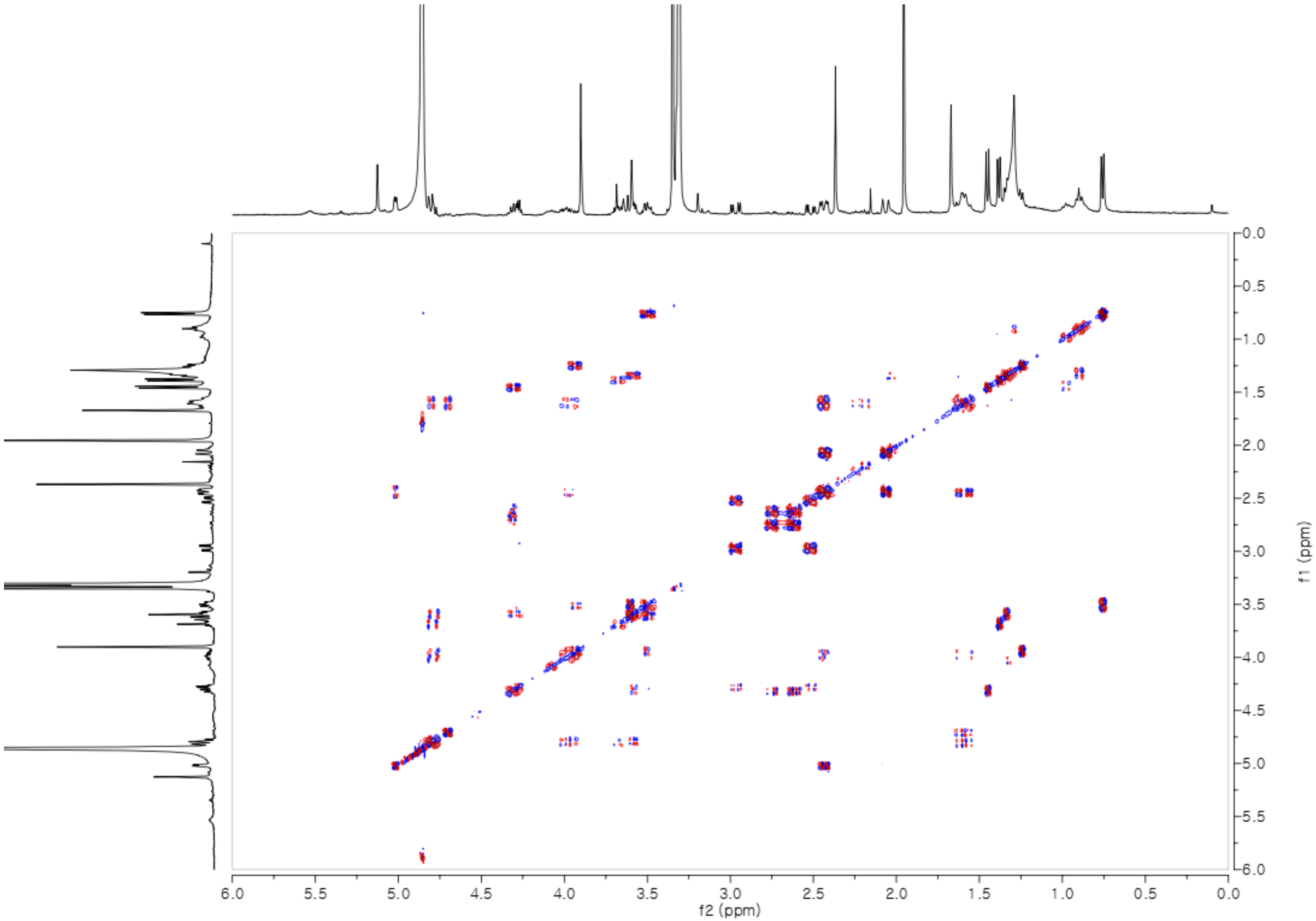

Figure S14. HSQC spectrum of **2** in methanol-d_4.

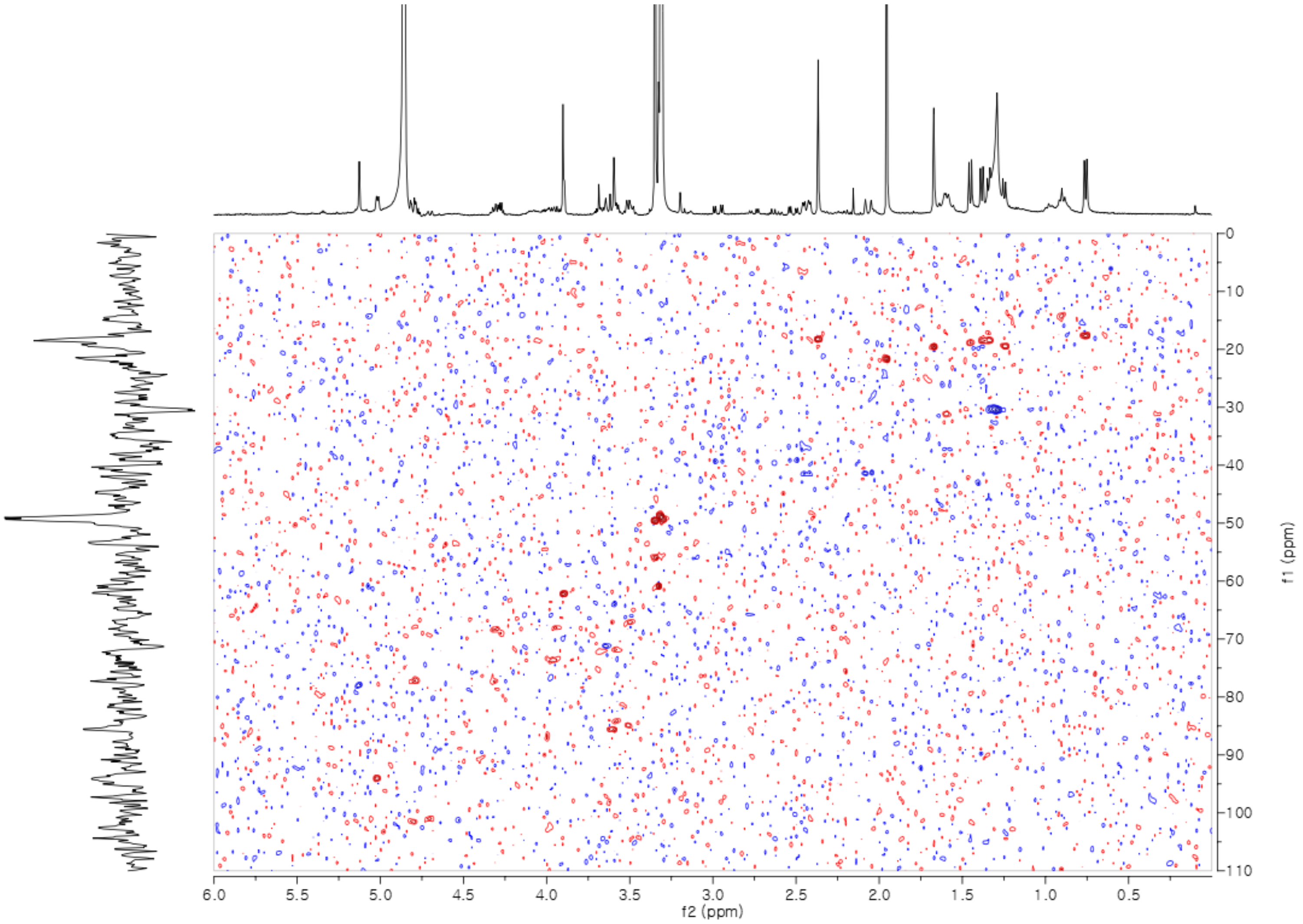

Figure S15. HMBC spectrum of **2** in methanol-d_4.

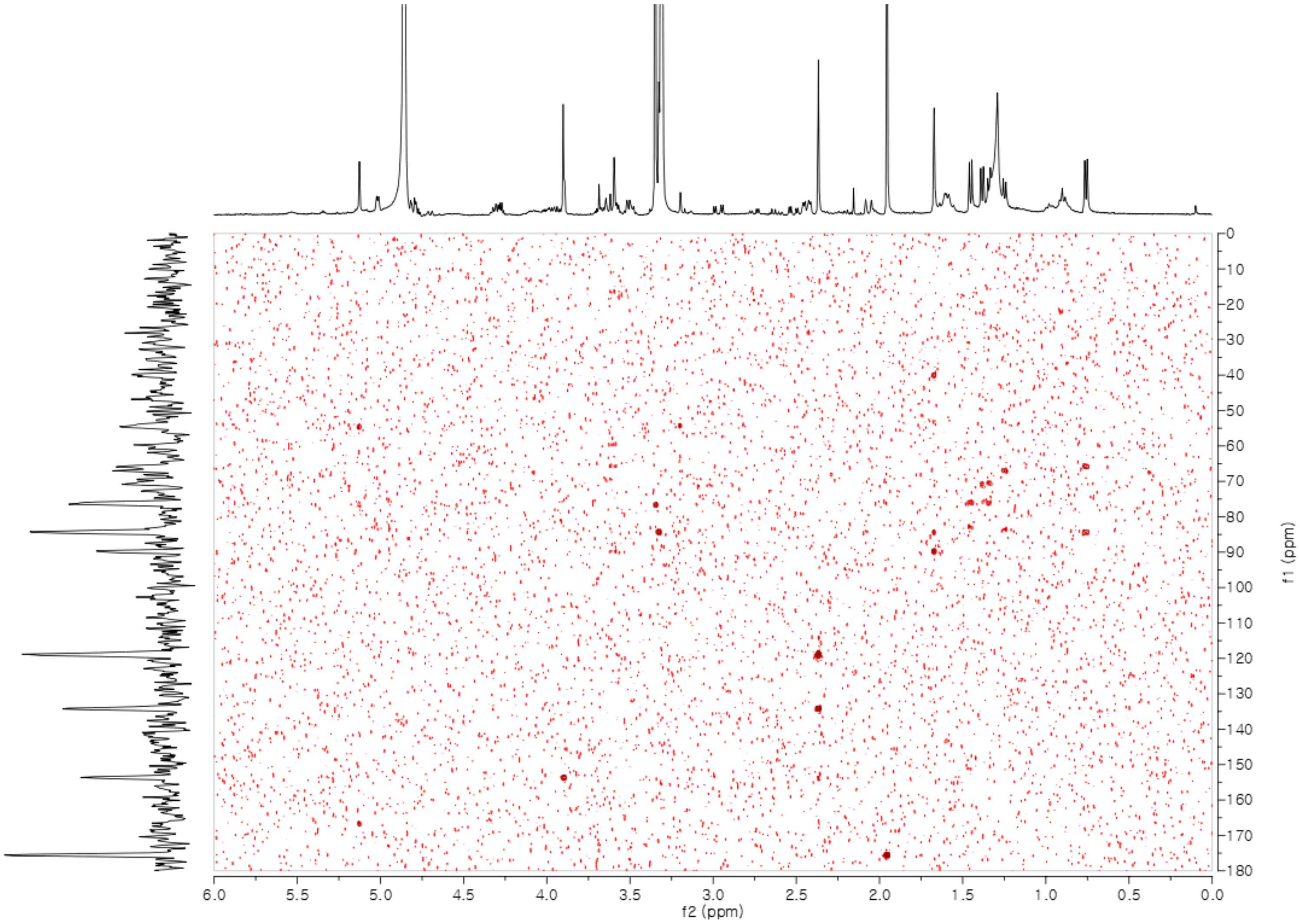

Figure S16. HRESIMS spectrum of **3**.

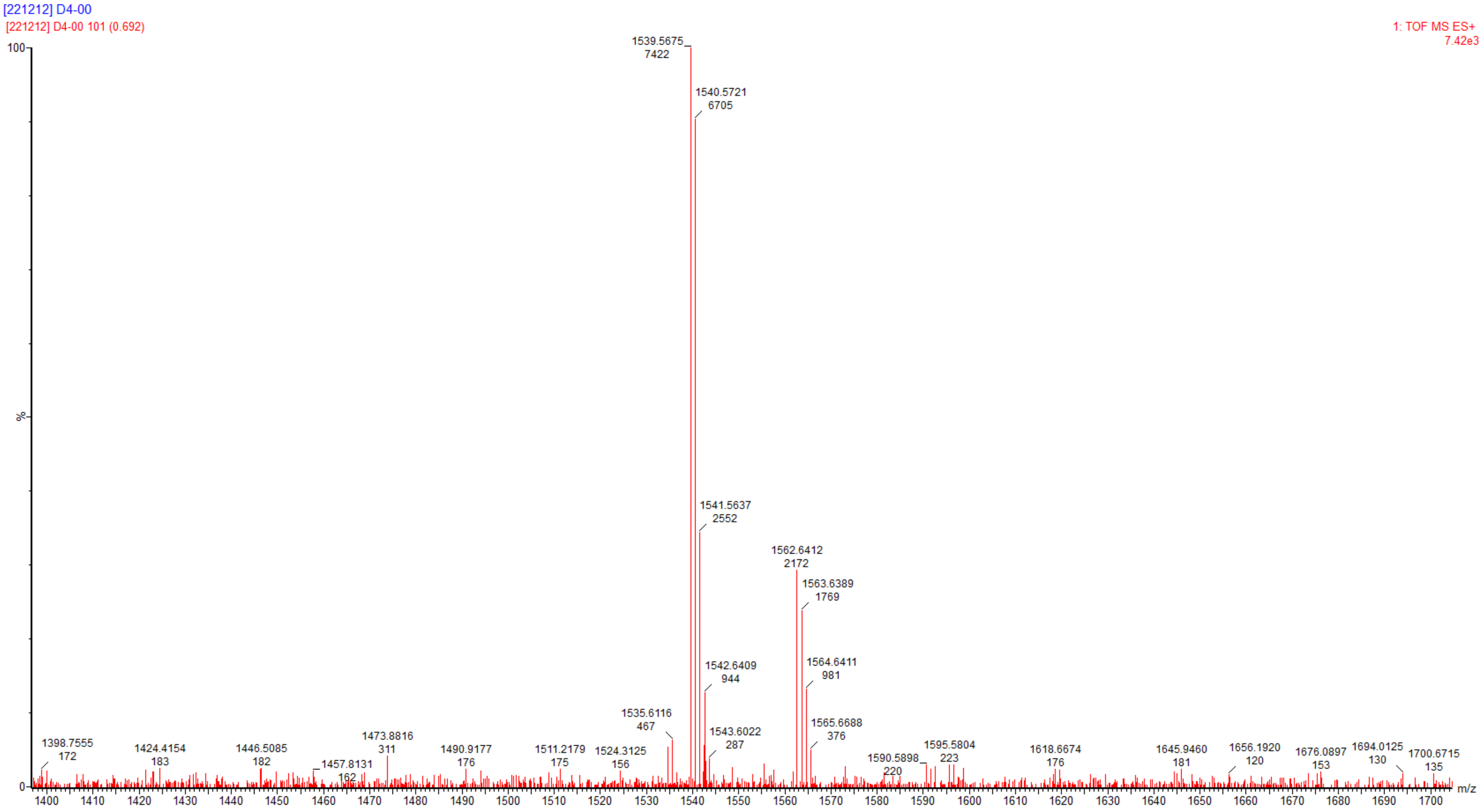

Figure S17. ^{1}H NMR spectrum of **3** in methanol-d_4.

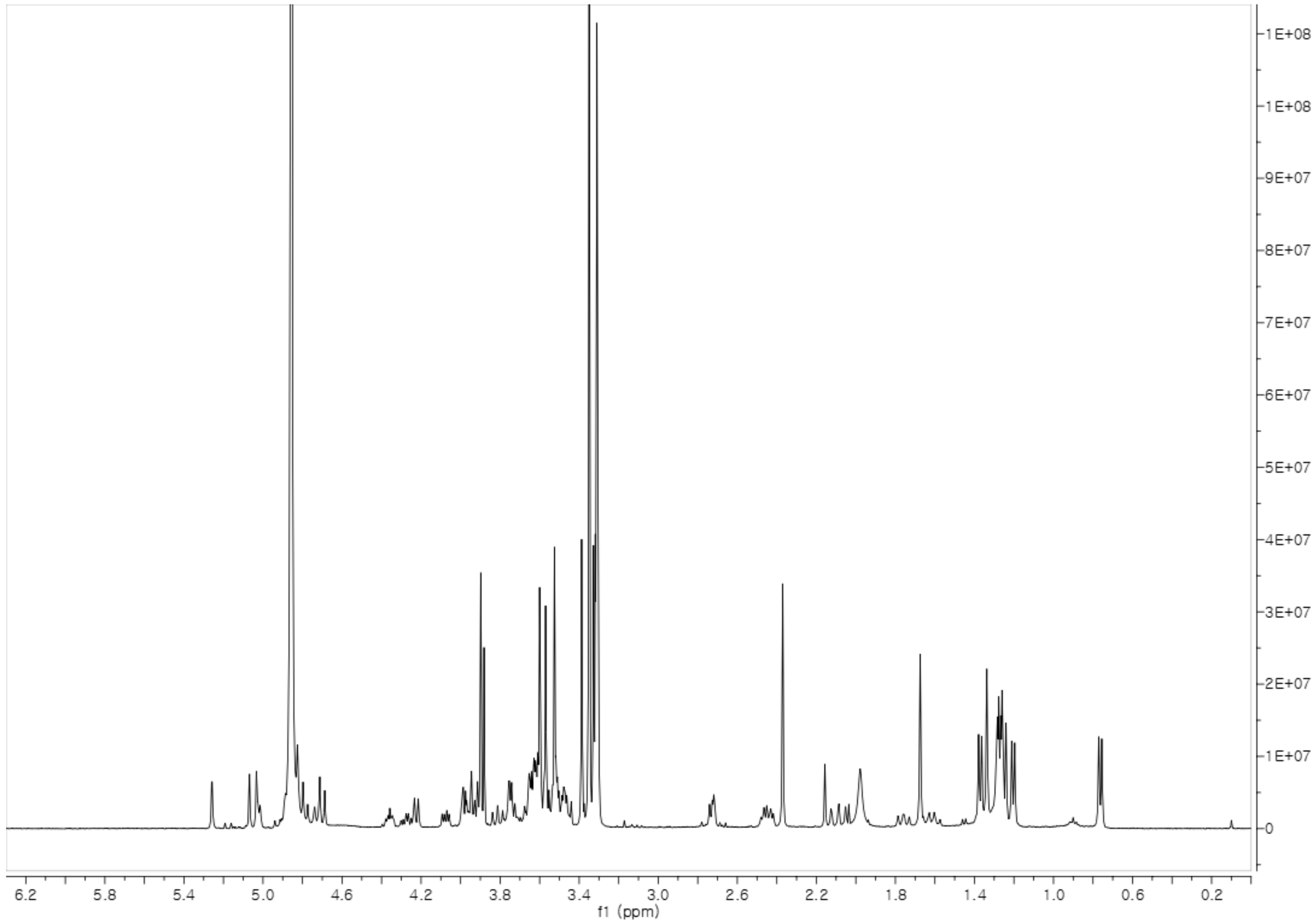

Figure S18. COSY spectrum of **3** in methanol-d_4.

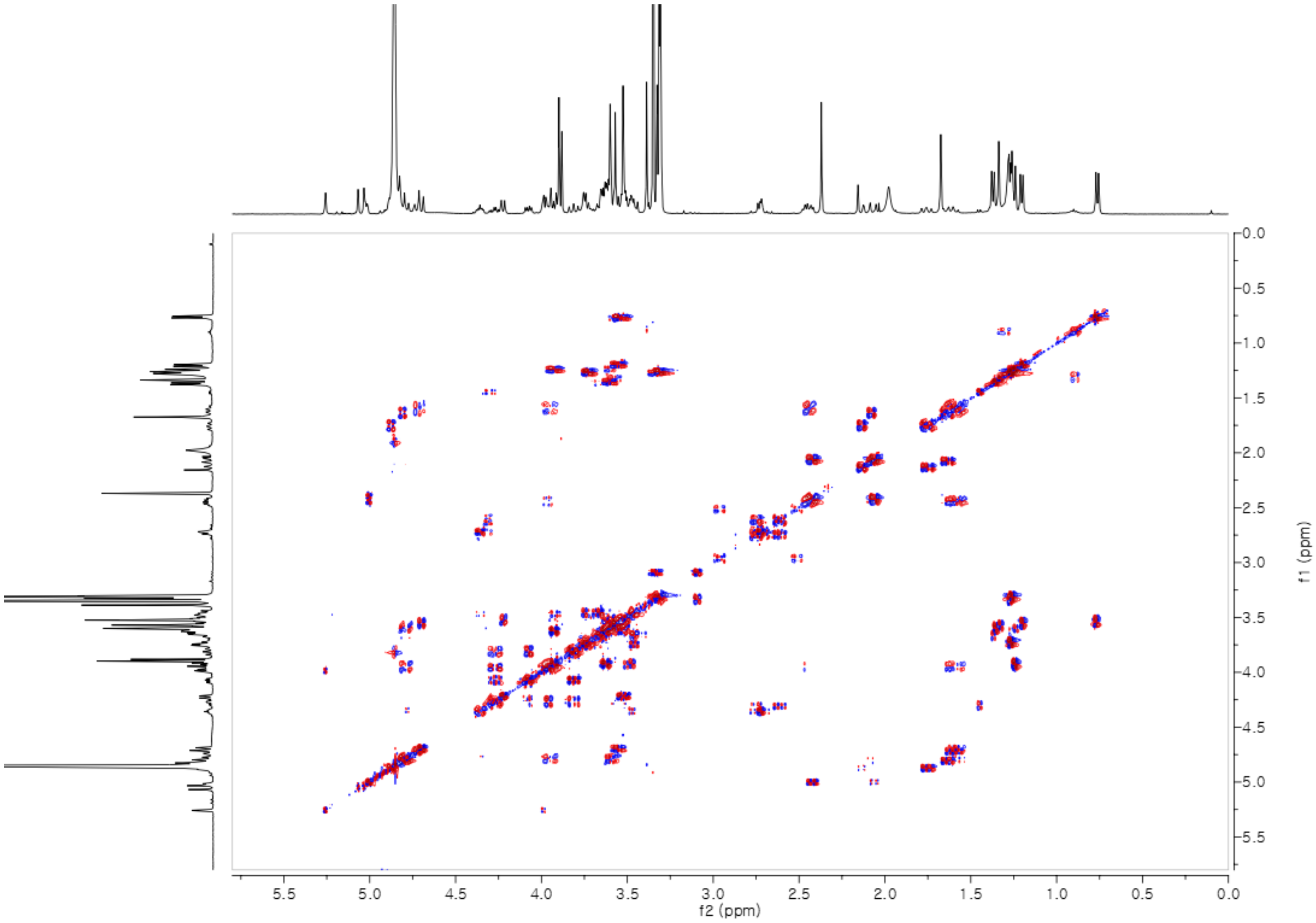

Figure S19. HSQC spectrum of **3** in methanol-d_4.

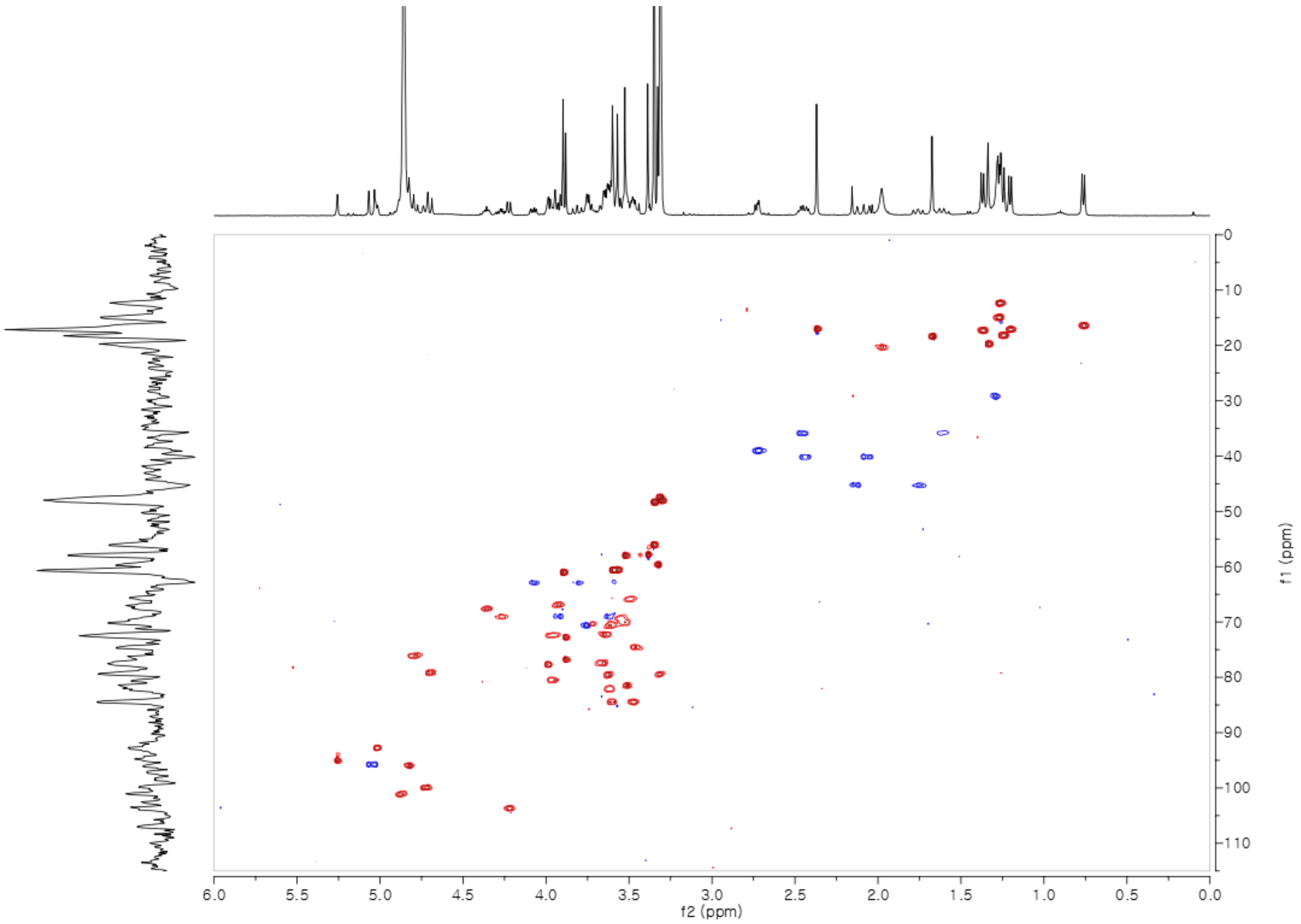

Figure S20. HMBC spectrum of **3** in methanol-d_4.

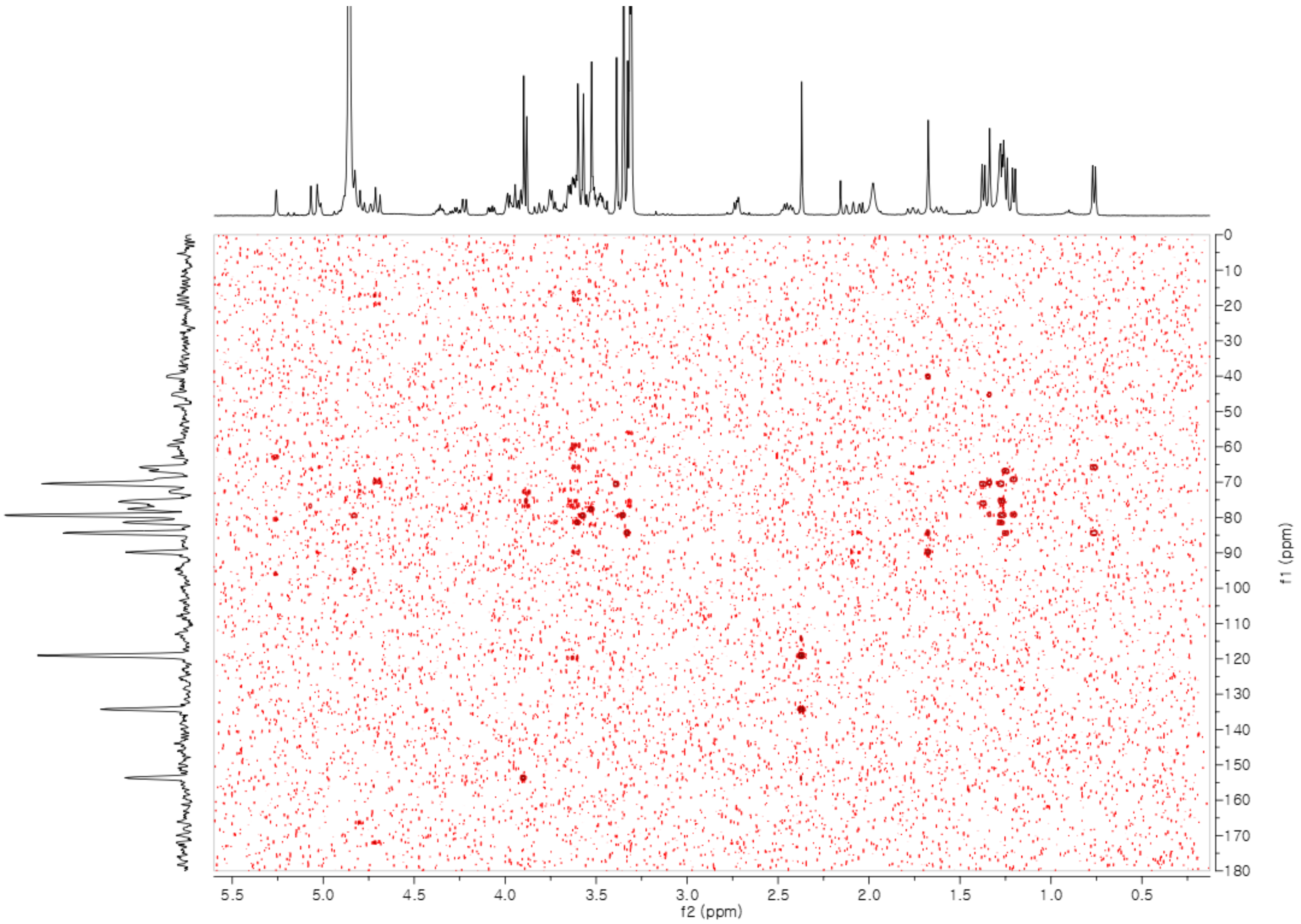

Figure S21. TOCSY spectrum of **3** in methanol-d_4.

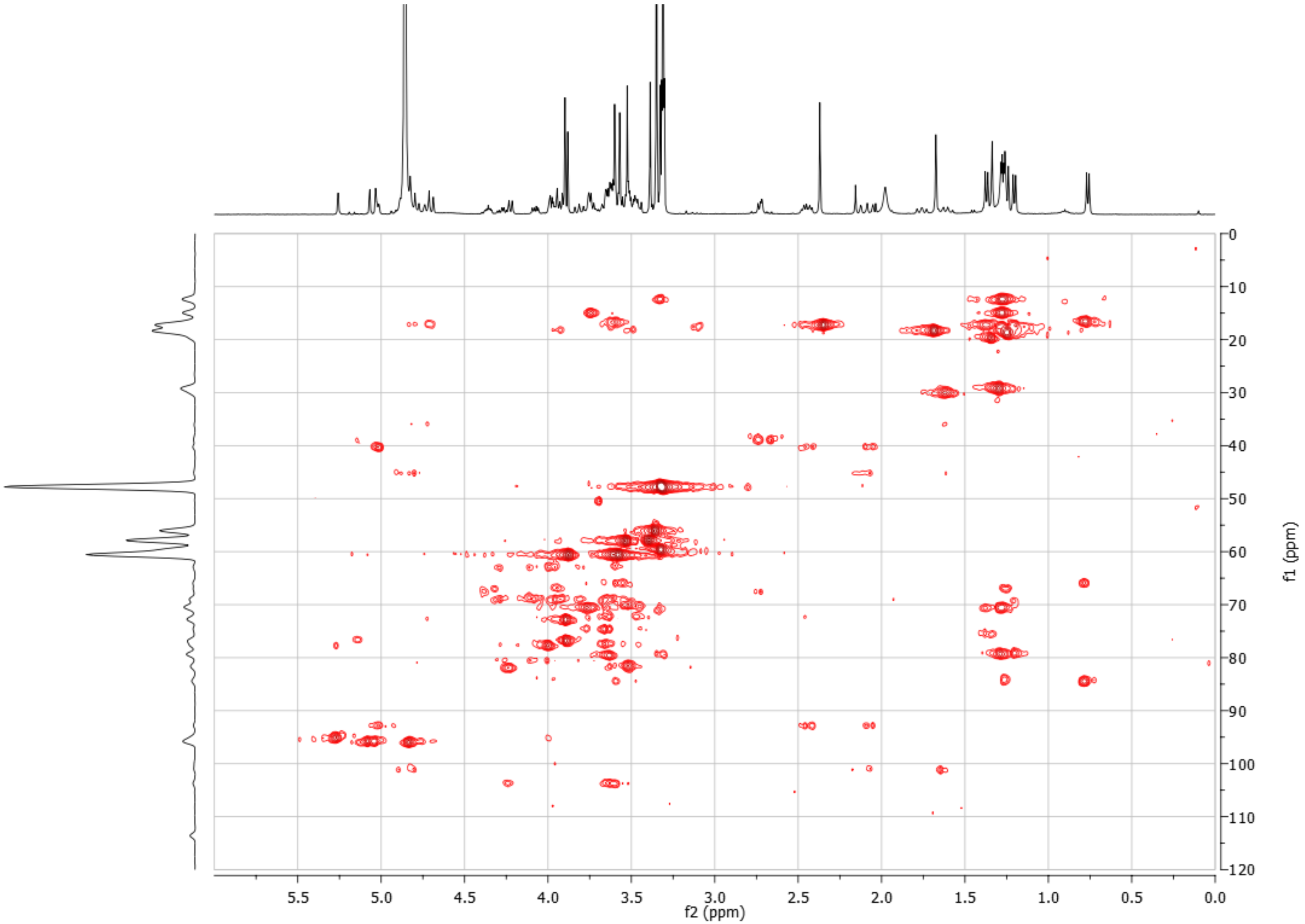

Table S1. ^{1}H (400 MHz) and ^{13}C (100 MHz) NMR Spectroscopic Data for Compound **3** in Methanol-d_4 (δ in ppm, J values in Hz).

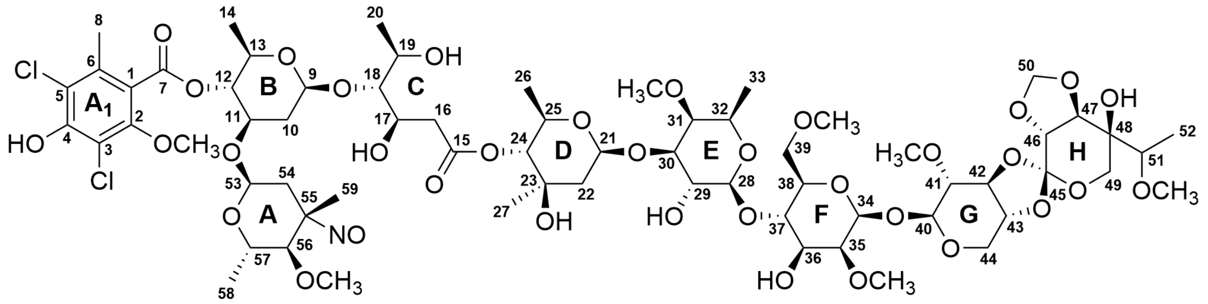

3

Position	3 δ_C	3 δ_H (J in Hz)	Position	3 δ_C	3 δ_H (J in Hz)
1	114.2		34	95.9	4.82, m
2	153.8		35	79.5	3.62, m
3	n.d.[a]		36	72.3	3.65, m
4	n.d.[a]		37	77.5	3.65, m
5	119.0		38	74.6	3.46, dd (6.1, 3.3)
6	134.3		39	70.6	3.75, d (4.8)
7	166.4		35-OCH_3	60.6	3.57, s
8	17.1	2.37, s	39-OCH_3	57.8	3.38, s
2-OCH_3	61.0	3.90, s	40	95.1	5.26, d (1.0)
9	99.9	4.73, m	41	77.7	3.99, m
10a	35.9	2.47, m	42	80.6	3.96, m
10b		1.62, m	43	69.0	4.27, m
11	72.4	3.96, m	44a	62.9	4.08, dd (9.6, 4.6)
12	76.2	4.79, d (9.4)	44b		3.81, m
13	70.6	3.62, m	41-OCH_3	58.0	3.52, s
14	17.3	1.37, d (6.2)	45	77.7	
15	171.8		46	72.8	3.88, m
16	39.0	2.73, m	47	76.8	3.88, m

17	67.5	4.36, ddd (8.0, 4.7, 2.3)	48	75.4	
18	84.4	3.48, dd (6.1, 3.3)	49a	69.0	3.93, m
19	66.9	3.93, m	49b		3.62, m
20	18.1	1.24, d (6.5)	50a	95.7	5.07, s
21	101.2	4.88, m	50b		5.03, s
22a	45.3	2.11, m	51	79.4	3.32, overlaped
22b		1.76, dd (12.5, 10.2)	52	12.3	1.27, d (2.6)
23	70.2		51-OCH_3	56.0	3.35, s
24	79.1	4.70, d (10.0)	53	92.8	5.02, d (4.3)
25	70.6	3.62, m	54a	40.2	2.43, m
26	17.2	1.20, d (6.1)	54b		2.06, m
27	19.7	1.34, s	55	89.8	
28	103.7	4.22, d (7.7)	56	84.5	3.59, m
29	69.4	3.52, m	57	65.9	3.50, m
30	82.3	3.61, m	58	16.4	0.76, d (6.1)
31	81.4	3.51, m	59	18.3	1.67, s
32	70.3	3.72, m	56-OCH_3	59.6	3.32, s
33	15.0	1.26, d (2.7)			
31-OCH_3	60.6	3.60, s			

[a] n.d. not detected.

Everninomicin G$_1$ (3): Pale brownish gum; UV (MeOH) λ_{max} 210 nm; ^{1}H MNR (400 MHz) and ^{13}C NMR (100 MHz) data in methanol-*d*$_4$, see Table 2; HRESIMS (positive-ion mode) *m*/*z* 1539.5675 $[M + 2H]^{2+}$ in the HRESIMS (calcd for $C_{66}H_{103}Cl_2NO_{35}^{2+}$, 1539.5677, error = 0.1 ppm).
